# Leucine zipper transcription factor-like 1 binds adaptor protein complex-1 and 2 and participates in trafficking of transferrin receptor 1

**DOI:** 10.1371/journal.pone.0226298

**Published:** 2020-01-02

**Authors:** Kanyarat Promchan, Ven Natarajan

**Affiliations:** Laboratory of Molecular Cell Biology, Leidos Biomedical Research, Inc., Frederick National Laboratory for Cancer Research, Frederick, MD, United States of America; Tohoku University, JAPAN

## Abstract

LZTFL1 participates in immune synapse formation, ciliogenesis, and the localization of ciliary proteins, and knockout of LZTFL1 induces abnormal distribution of heterotetrameric adaptor protein complex-1 (AP-1) in the *Lztfl1*-knockout mouse photoreceptor cells, suggesting that LZTFL1 is involved in intracellular transport. Here, we demonstrate that *in vitro* LZTFL1 directly binds to AP-1 and AP-2 and coimmunoprecipitates AP-1 and AP-2 from cell lysates. DxxFxxLxxxR motif of LZTFL1 is essential for these bindings, suggesting LZTFL1 has roles in AP-1 and AP-2-mediated protein trafficking. Since AP-1 and AP-2 are known to be involved in transferrin receptor 1 (TfR1) trafficking, the effect of LZTFL1 on TfR1 recycling was analyzed. TfR1, AP-1 and LZTFL1 from cell lysates could be coimmunoprecipitated. However, pull-down results indicate there is no direct interaction between TfR1 and LZTFL1, suggesting that LZTFL1 interaction with TfR1 is indirect through AP-1. We report the colocalization of LZTFL1 and AP-1, AP-1 and TfR1 as well as LZTFL1 and TfR1 in the perinuclear region (PNR) and the cytoplasm, suggesting a potential complex between LZTFL1, AP-1 and TfR1. The results from the disruption of adaptin recruitment with brefeldin A treatment suggested ADP-ribosylation factor-dependent localization of LZFL1 and AP-1 in the PNR. Knockdown of AP-1 reduces the level of LZTFL1 in the PNR, suggesting that AP-1 plays a role in LZTFL1 trafficking. Knockout of LZTFL1 reduces the cell surface level and the rate of internalization of TfR1, leading to a decrease of transferrin uptake, efflux, and internalization. However, knockout of LZTFL1 did not affect the cell surface levels of epidermal growth factor receptor and cation-independent mannose 6-phosphate receptor, indicating that LZTFL1 specifically regulates the cell surface level of TfR1. These data support a novel role of LZTFL1 in regulating the cell surface TfR1 level by interacting with AP-1 and AP-2.

## Introduction

Leucine zipper transcription factor-like 1 (LZTFL1) is a cytoplasmic and ciliary protein that interacts with other cytosolic proteins, regulates cilia trafficking, and controls β-catenin nuclear localization [1–4]. LZTFL1 is induced by all-*trans* retinoic acid in activated T cells and associates with the immune synapse [5]. LZTFL1 inhibits lung tumorigenesis, possibly by maintaining epithelial cell differentiation or inhibition of signaling, leading to epithelial–mesenchymal transition [3]. Defects in LZTFL1 are associated with mesoaxial polydactyly, polydipsia, polyuria, and massive enlarged cystic kidneys [6] and retinal degeneration [7]. LZTFL1, recently designated as a Bardet-Biedl syndrome protein (BBS), BBS17, binds to BBS9, a constituent of the BBSome complex, and regulates ciliary localization of the BBSome [1]. Knockout of LZTFL1 altered the localization of many proteins of the photoreceptor outer segment [8]. We have shown that adaptor protein complex-1 (AP-1) was abnormally distributed in the *Lztfl1*-knockout mouse photoreceptor cells, suggesting that LZTFL1 has a role in AP-1-mediated protein trafficking [7].

Five types of adaptor protein complexes (APs), AP-1 to AP-5, have been identified as parts of the coated vesicle [9]. They are heterotetramers composed of two large subunits (β1–β5 and γ, α, δ, ε, or ζ), one medium subunit (μ1–μ5), and one small subunit (σ1–σ5) [10, 11]. Among the five APs, AP-1 and AP-2 are well characterized as clathrin adaptor protein complexes for membrane proteins trafficking from the trans-Golgi network (TGN) to endosomes leading to plasma membrane sorting and recycling from the plasma membrane to endosomes, respectively [12, 13]. The β2 subunit of AP-2, which shares high protein identity with the β1 subunit of AP-1, is known to interact with accessory proteins to transport cargo proteins to target compartments [14–16]. Previous studies have suggested that AP-1 facilitates transferrin receptor (TfR1) trafficking from the TGN to the cell membrane [17–20]. Here, we identify AP-1 and AP-2 as LZTFL1-interacting proteins and show that these interactions play an important role in membrane protein trafficking. Data presented here suggest that LZTFL1 functions as an accessory protein for AP-1 and AP-2 and regulates the cell surface level of TfR1.

## Materials and methods

### Plasmids and antibodies

Plasmids expressing human LZTFL1 (NM_020347) and TfR1 (NM_003234) with Myc-FLAG tag in pCMV6-Entry vector were purchased from OriGene Technologies (Rockville, MD). The following LZTFL1 mutants were generated by substituting conserved amino acids with alanine: DFLR (DxxFxx[L/F] xxxR motif mutation), DLM (dileucine-based motif mutation), LZM (leucine zipper motif mutation), and SKR (SKR mutation) (S2 Table). The SNARE domain, leucine zipper motif, and coiled-coil domain of LZTFL1 were deleted to obtain mutants ΔSNARE, ΔLZM, and ΔCC, respectively, while the deletion of amino acids 11–146 of LZTFL1 resulted in ΔNT. All LZTFL1 mutants were subcloned into a pCMV6-Entry vector (AMS Biotechnology, Abingdon, United Kingdom). All plasmids were verified by DNA sequencing. Plasmids expressing GFP-FLAG (pEGFP-N1-FLAG, 60360) and AP2B1 (Myc-DDK-tagged human adaptor protein complex 2, beta 1 subunit, RC201129) were purchased from Addgene (Watertown, MA).

Plasmids expressing maltose-binding protein (MBP) fused with human LZTFL1 and mutants were generated by cloning in a pMAL-c2X vector (New England Biolabs, Inc., Ipswich, MA). Coding sequences for human β1, γ, μ1, and σ1 subunits of AP-1 from HEK293FT cell cDNA and β2 subunit of AP-2 of Myc-DDK-tagged AP2B1 plasmid, were PCR-amplified and subcloned into a pGEX-6P-1 vector (GE Healthcare, Chicago, IL) to obtain glutathione S-transferase (GST)-fused β1, γ, μ1, and, σ1 subunits of AP-1 and β2 subunit of AP-2 expression plasmids, respectively.

Antibody to LZTFL1 (HPA043466, 1:200 for immunofluorescence, 1:2500 for western blot and immunoprecipitation) was purchased from Atlas Antibodies (Bromma, Sweden). Antibodies against TfR2 (MA5-25928, 1:500 for western blot and 1:100 for immunofluorescence) was purchased from Thermo Fisher Scientific (Waltham, MA). Antibodies to LZTFL1 (sc-100968, 1:50 for immunofluorescence; sc-100968 and sc-376022, 1:1000 for western blot), GST (sc-138, 1:5000 for western blot), α-adaptin 1 (AP-2, sc-10761, for immunoprecipitation, 1:1000 for western blot), tubulin (sc-8035, 1:10000 for western blot), actin (sc-8432, 1:1000 for western blot) and normal mouse IgG (sc-2025) were obtained from Santa Cruz Biotechnology, Inc. (Dallas, TX). FLAG-M2 magnetic beads as well as antibodies to the γ subunit of AP-1 (A4200, for immunoprecipitation, 1:200 for immunofluorescence and 1:1000 for western blot), the β1 and β2 subunits of AP-1 and AP-2 (A4450, 1:1000 for western blot), β-actin (A1978, 1:10000 for western blot) were purchased from Sigma (St. Louis, MO). Anti-FLAG monoclonal (DDK, TA50011, 1:5000 for western blot) and anti-MBP (E8032S, 1:5000 for western blot) antibodies were purchased from OriGene and New England Biolabs, respectively. Antibodies against TGN marker p230 (611280, 1:250 for immunofluorescence) and epidermal growth factor receptor (EGFR, 610017, 1:1000 for western blot) were obtained from BD Biosciences (San Jose, CA). Antibodies to TfR1 (ab84036, 1:200 for immunofluorescence and 1:5000 for western blot) and cation-independent mannose-6-phosphate receptor (CI-MPR, ab8093, 1:1000 for western blot) were purchased from Abcam (Cambridge, MA). Alexa-conjugated secondary antibodies used for immunofluorescence staining were purchased from Invitrogen (Waltham, MA). All fluorescence-conjugated secondary antibodies used in western blot analysis were purchased from LI-COR (Lincoln, NE).

### The identification of LZTFL1-interacting proteins

Jurkat E6.1 cell line was cultured in RPMI-1640 (Thermo Fisher Scientific) as previously described [21] and transfected with plasmids expressing LZTFL1-Myc-Flag or pCMV6-Entry vector (Origene) by electroporation (BioRad) and cultured for 48 hours. The FLAG-fusion proteins were immunoprecipitated by incubating with anti-FLAG M2 magnetic beads (Sigma) overnight at 4°C. The beads were washed three times with lysis buffer, and the bound proteins were eluted with 3X FLAG peptide (Sigma). Eluted proteins were incubated with NuPAGE LDS sample buffer (Thermo Fisher Scientific) at 99°C for 10 minutes then separated on 4–12% gradient SDS-PAGE (Thermo Fisher Scientific).

Protein gel was stained with Pierce silver stain for mass spectrometry (Thermo Fisher Scientific) according to the manufacturer’s protocol. In-gel digestion and mass spectrometry analysis were carried out as previously described [22].

### Protein purification and pull-down

GST-fused and MBP-fused proteins were purified from *E*. *Coli* by affinity chromatography with Glutathione Sepharose 4B and amylose resin, respectively, as described in the instruction manuals (GE Healthcare and New England Biolabs, respectively). Purified GST-fused and MBP-fused proteins were dialyzed using Slide-A-Lyzer dialysis cassettes (Thermo Fisher Scientific) and were concentrated by Amicon Ultra Centrifugal filter devices (Millipore, Billerica, MA) and were separated by electrophoresis on a 10% SDS-PAGE and visualized by Coomassie Brilliant Blue (CBB) staining (Simply Blue SafeStain, Thermo Fisher Scientific).

For direct interaction studies, MBP-fused proteins were incubated with amylose resin at 4°C for 2 hours and washed with PBS. Purified GST-fused proteins were added to amylose-resin-bound MBP-fused proteins, and the mixture was incubated overnight at 4°C. After washing, the bound proteins were eluted with NuPAGE LDS sample buffer at 99°C for 10 minutes.

Twenty five microgram of purified MBP-LZTFL1 fusion protein was cleaved in 20 mM Tris-HCl, 100 mM NaCl, 2 mM CaCl_2_ (pH 8.0) containing 1 μg of Factor Xa (New England Biolabs) for 6 hours at room temperature and used for direct interaction studies with purified TfR1-Myc-Flag protein (TP326147, OriGene) conjugated anti-TfR1 antibody beads or GST-fused β1subunit of AP-1 conjugated Glutathione Sepharose 4B.

### Generation of *Lztfl1*-knockout mice

*Lztfl1*-knockout mice were generated as previously described [7].

### Co-immunoprecipitation

HEK293FT cells (R70007, Thermo Fisher Scientific) were cultured in DMEM containing 2 mM glutamine, 10% FBS, and 1% penicillin-streptomycin (complete DMEM). Cells were transfected with FLAG, GFP-FLAG, wild-type-LZTFL1-FLAG or mutant-LZTFL1-FLAG expressing plasmids for 24 hours using Lipofectamine 2000 (Invitrogen), then lysed with lysis buffer containing 30% glycerol. The FLAG-fusion proteins were immunoprecipitated by anti-FLAG M2 magnetic beads and eluted with 3X FLAG peptide.

For co-immunoprecipitation of HEK293FT endogenous proteins, HEK293FT cells were lysed in lysis buffer containing 30% glycerol. Protein A/G magnetic beads (Thermo Fisher Scientific) conjugated with normal mouse IgG, anti-γ subunit of AP-1 and anti-α subunit of AP-2 were prepared by incubating the beads and normal mouse IgG or antibodies in coupling buffer (Thermo Fisher Scientific) at room temperature for 30 minutes. The beads were then washed three times with lysis buffer. The lysate was immunoprecipitated and the bound proteins were eluted with elution buffer (Thermo Fisher Scientific).

For co-immunoprecipitation of mouse endogenous proteins, brain tissues from wild-type and *Lztfl1*-knockout mice were homogenized in lysis buffer containing 30% glycerol using 7 ml Dounce homogenizer (Wheaton, Millville, NJ). The tissue lysate was immunoprecipitated with anti-LZTFL1-conjugated magnetic beads, and the bound proteins were eluted with elution buffer.

### Brefeldin A treatment

HeLa cells obtained through the NIH AIDS Reagent Program, Division of AIDS, NIAID, NIH (Cat #154) [23] were seeded in poly-L-lysine-coated 8-well chamber slides for 36 hours and treated with either 7.5 μg/ml brefeldin A (BFA) (Sigma) or DMSO in complete DMEM at 37°C and 5% CO_2_ for 2 minutes. After washing three times with complete DMEM, cells were incubated with complete DMEM at 37°C and 5% CO_2_ for 0, 15, 30, or 60 minutes and then fixed and immunostained with the indicated antibodies.

### AP-1 knockdown by RNA interference

HeLa cells were transfected with 25 nM control siRNA or AP-1 siRNA (ON-TARGETplus Human AP1G1 siRNA-SMART pool, Dharmacon, Lafayette, CO) by reverse transfection using Lipofectamine 2000 as specified by the manufacturer in either fibronectin-coated 8-well chamber slides for immunofluorescence staining or in 6-centimeter dishes for western blot analysis. After 16 hours, cells were washed with complete DMEM once and transfected again with control siRNA or AP-1 siRNA for 24 hours. Cells were then fixed and immunostained with the indicated antibodies for immunofluorescence or harvested for western blot analysis.

### Establishment of *LZTFL1*-knockout HeLa cells

*LZTFL1*-knockout HeLa cells were generated via the CRISPR/Cas9-based genome-editing method using the pCas-Guide plasmid and GFP-puromycin donor plasmid. Two LZTFL1 gRNA sequences (5’-CCCCCTATGTACGCCTCCC-3’ and 5’-CTTTGACAAACTTGACTTT-3’) cloned in pCAS-Guide-eGFP vector and a GFP-puromycin plasmid with homologous arms to LZTFL1 (donor DNA) were purchased from Origene (Rockville, MD). The plasmids were transfected into HeLa cells with Lipofectamine 2000, and cells were selected with 1.0 μg/mL of puromycin for a week and individual clones were isolated by limited dilution. *LZTFL1*-knockout clones were screened by PCR for insertion of puromycin resistant gene in *LZTFL1*. Biallelic *LZTFL1* knockout were verified by immunoblotting for LZTFL1.

### Quantitation of cell surface level of TfR1, EGFR and CI-MPR

Wild-type and *LZTFL1*-knockout HeLa cells were seeded in 10-centimeter dishes for 24 hours. Cells were washed twice with cold PBS and incubated with 2 mM Sulfo-NHS-LC-Biotin (Thermo Fisher Scientific) in cold PBS at 4°C for 30 minutes. Excess NHS groups were quenched by incubating three times with 100 mM glycine for 5 minutes each, then washing twice with PBS. Cells were lysed at 4°C for 40 minutes. Protein supernatants were mixed with 30 μl of streptavidin magnetic beads (Thermo Fisher Scientific) at room temperature for 2 hours with gentle rotation. After washing the beads three times with the lysis buffer followed by PBS, biotinylated proteins were eluted with NuPAGE LDS sample buffer with DTT for TfR1 and EGFR or without DTT for CI-MPR at 99°C for 10 minutes and western blotted with antibodies against TfR1, EGFR and CI-MPR. The cell surface level of TfR1, EGFR and CI-MPR was calculated by the band intensity of biotinylated protein versus total protein.

### Estimation of the rate of TfR1 internalization

Wild-type and *LZTFL1*-knockout HeLa cells were seeded in 10-centimeter dishes for 24 hours. Cells were washed twice with cold PBS and incubated with 2 mM Sulfo-NHS-SS-Biotin (Thermo Fisher Scientific) in cold PBS at 4°C for 30 minutes. Excess NHS groups were quenched by incubating three times with 100 mM glycine for 5 minutes each, then washing three times with PBS. Cells were incubated with warm complete DMEM at 37°C for the indicated time to allow the internalization of membrane proteins, then washed twice with cold NT buffer (20 mM Tris-HCl pH 8.0, 150 mM NaCl, 1 mM EDTA and 0.2% BSA). The remaining biotin-labeled cell surface proteins were stripped three times with cold 100 mM Mesna (Sigma) in NT buffer at 4°C for 20 minutes each time, then washed twice with cold NT buffer. Cells were incubated with ice-cold 120 mM iodoacetamide (Sigma) in NT buffer at 4°C for 10 minutes to quench the remaining Mesna, then washed three times with PBS. Cells were lysed at 4°C for 40 minutes. Lysates were mixed with 30 μl of streptavidin magnetic beads (Thermo Fisher Scientific) at 4°C for 2 hours with gentle rotation. After washing the beads three times with the lysis buffer followed by PBS, biotinylated proteins were eluted with NuPAGE LDS sample buffer at 99°C for 10 minutes and western blotted with antibody against TfR1. The rate of TfR1 internalization was calculated from the band intensity of the biotinylated TfR1 at 10 and 20 min after biotin strip to the biotinylated TfR1 at 0 min before the biotin strip.

### Immunoblot analysis

Proteins in cell lysates and immunoprecipitates in the NuPAGE LDS sample buffer were separated on either 4%-12% gradient or 10% SDS-PAGE and transferred onto a polyvinylidene difluoride membrane (Millipore). Blots were stained with REVERT Total Protein Stain (LI-COR) to verify equal loading and transfer of proteins, then blocked with Odyssey blocking buffer (LI-COR) at room temperature for 1 hour and incubated overnight at 4°C with the indicated antibodies in the Odyssey blocking buffer containing 0.05% TWEEN 20. After washing with PBS containing 0.05% TWEEN 20, the membrane was incubated with fluorescent-dye-labeled goat anti-mouse-IgG or goat anti-rabbit-IgG secondary antibodies (LI-COR). Immunoreactive protein bands were visualized using the LI-COR detection system. Immunoblots represent data from three independent experiments.

### Immunofluorescence

HeLa cells were cultured in complete DMEM and seeded in poly-L-lysine-coated or fibronectin-coated 8-well chamber slides for 24 hours and processed for staining for endogenous proteins. Cells were fixed with 4% formaldehyde at room temperature for 20 minutes and washed twice with PBS, then incubated with 5% goat serum or 4% FBS in PBS containing 0.2% triton X-100 or 5% goat serum in PBS without triton X-100 (for cell surface TfR2 staining) at room temperature for 2 hours. Cells were immunostained with a primary antibody at 4°C overnight. After washing three times with PBS, cells were incubated at room temperature for 1 hour with goat anti-mouse or goat anti-rabbit secondary antibodies conjugated with either Alexa 488, Alexa 568, or Alexa 647 (Life Technologies), then washed three times with PBS. To stain with an additional antibody for a different protein, cells were blocked again with 5% goat serum at room temperature for 2 hours, and the staining was repeated as above. Cells were then mounted with ProLong® Antifade Mountant with DAPI (Life Technologies).

### Estimation of transferrin uptake, efflux, and internalization

Wild-type and *LZTFL1*-knockout HeLa cells were seeded in poly-L-lysine-coated or fibronectin-coated 8-well chamber slides for 24 hours. Cells were rinsed with PBS and incubated in serum-free DMEM for 30 minutes, then washed twice with PBS. For transferrin uptake, cells were incubated with 25 μg/ml transferrin conjugated to Alexa Fluor 568 (Tfn-568; Life Technologies) in serum-free media at 37°C for the indicated times, which was followed by fixation. For transferrin efflux, cells were incubated with 25 μg/ml Tfn-568 in serum-free DMEM at 37°C for 30 minutes, washed with PBS three times, and fixed immediately (time point 0), or they were incubated with complete DMEM at 37°C for the indicated times and then rinsed and fixed. For transferrin internalization, cells were incubated with 25 μg/ml ice-cold Tfn-568 in serum-free DMEM at 4°C for 30 minutes. Cells were washed with ice-cold PBS three times and fixed immediately (time point 0), or they were incubated with complete DMEM at 37°C for the indicated times and then rinsed and fixed.

### Confocal microscopy and immunofluorescence image analysis

Immunofluorescence images were acquired by LSM 510 and LSM 710 confocal microscopes (Zeiss) with a Plan-Apochromat 63X/1.4 Oil DIC objective lens, 512 x 512 pixels image size, 8 bit depth. Samples labeled with Alexa 488, Alexa 568, Alexa 647, and DAPI were excited with 488 nm, 561 nm, 633 nm, and 405 nm lasers, respectively.

The immunofluorescence images were acquired and analyzed by LSM 510 and Fiji visualization and analysis software (ImageJ, version 1.51). Target protein level was quantitated using default measurement as integrated density by Fiji. Using plug-ins embedded in Fiji, colocalization analysis was performed on a similar-sized symmetrical region of interest (ROI) selected for each dye. Background levels were subtracted from each ROI before further analysis. Each colored image was split into respective red, green, magenta, and blue channels. The colocalization images were analyzed using the RG2B colocalization plug-in to further visualize the colocalized pixels rendered as white, after which the intensity of colocalized pixels per ROI was measured. The comparative degree of colocalization for target proteins was calculated as the mean of Pearson’s *R* coefficient and Costes Significance Test [24] (with Costes randomization = 200) on the red and green channels using the embedded colocalization analysis plug-in (Coloc 2) at default settings. Pearson’s correlation coefficient was used to compare the relative degree of colocalization of target proteins between sample groups.

### Statistical analysis

Statistical analysis was conducted by a two-tailed t-test. Results are shown as mean ± the standard deviation. A p value of <0.01 was considered statistically significant and p values of <0.0001 is denoted in the figures by ***.

### Ethics statement

Frederick National Laboratory for Cancer Research is accredited by AAALAC International and follows the Public Health Service Policy for the Care and Use of Laboratory Animals. Animal care was provided in accordance with the procedures outlined in the *Guide for Care and Use of Laboratory Animal*s (National Research Council, 1996, National Academies Press: Washington, D.C.) The study involving animals was conducted according to relevant national and international guidelines for the care and use of experimental animals and was approved by the National Cancer Institute at Frederick Animal Care and Use Committee.

## Results

### Identification of an AP-1 subunit β-binding domain in LZTFL1

Knowledge of LZTFL1-interacting proteins is critical to gain insight into LZTFL1 functions. To this end, a proteomic analysis based on liquid chromatography–tandem mass spectrometry was carried out [25]. The β1 subunit of AP-1 and the clathrin heavy chain were among the proteins identified as LZTFL1-interacting proteins (S1 Table). AP-1 has been found to regulate cilium formation, axonemal length, and microtubule arrangement in *C*. *elegans*, and AP-1 knockdown in human cells can impair cilium structure, orientation, and microtubule post-translational modification [26]. Since LZTFL1 has been shown to have a role in ciliogenesis and protein transport in cilia, characterization of the interaction between LZTFL1 and the AP-1 complex was of interest.

**Fig 1:**
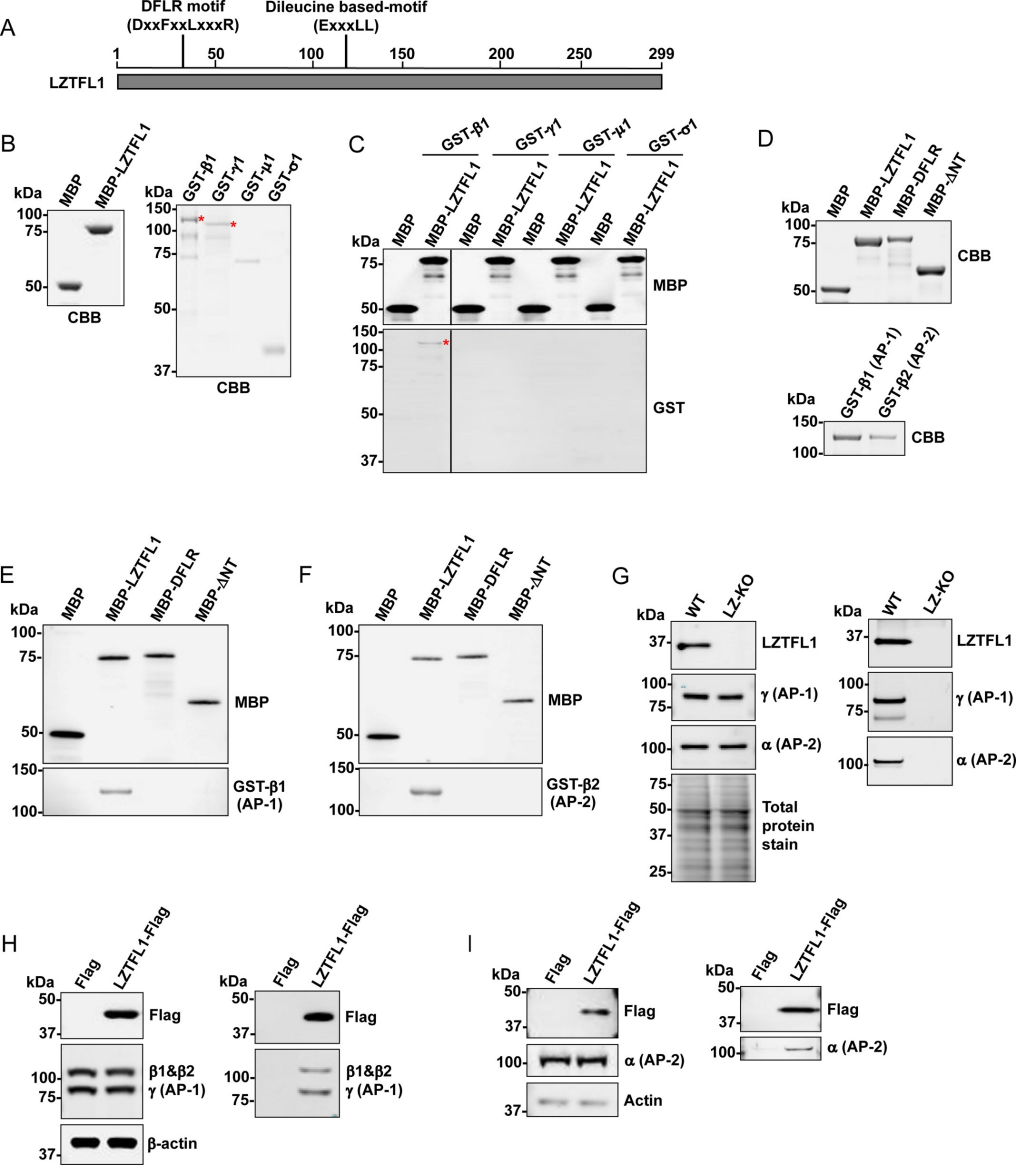
LZTFL1 directly binds to β1 subunit of AP-1 and β2 subunit of AP-2 *in vitro*. **(A)** Schematic representation of potential protein-binding domains of LZTFL1. **(B)** Purified LZTFL1 fused with maltose-binding protein (MBP, **left**) as well as individual subunits of AP-1 complex (β1, γ, μ1, and σ1) fused with glutathione S-transferase (GST, **right**) were visualized by Coomassie Brilliant Blue (CBB) staining. Full-length GST-β1 and GST-γ are indicated with red asterisks. **(C)** Purified MBP or MBP-LZTFL1 was incubated with each of the purified GST-fused subunits of the AP-1 complex (β1, γ, μ1, and σ1), and the LZTFL1-bound subunit was purified by amylose resin and analyzed by western blotting using anti-MBP and anti-GST antibodies. The GST-β1 band is indicated with a red asterisk. **(D, top)** Purified wild-type LZTFL1 and LZTFL1 mutants fused with MBP and **(D, bottom)** β1 and β2 subunits of AP-1 and AP-2 respectively fused with GST were visualized by CBB staining. Purified MBP, MBP-LZTFL1, or mutants were incubated with **(E)** GST-β1 (AP-1) or **(F)** GST-β2 (AP-2) and pulled-down proteins were analyzed by western blotting using anti-MBP and anti-GST antibodies. **(G)** Brain tissue lysate from wild-type and *Lztfl1*-knockout mice was analyzed by western blotting using anti-LZTFL1 antibody, anti-γ subunit of AP-1 antibody, anti-α subunit of AP-2 antibody, and REVERT staining for total protein **(left)**. Lysate was immunoprecipitated with anti-LZTFL1-conjugated beads. The LZTFL1-interacting proteins were eluted by NuPAGE LDS buffer and analyzed by western blotting using anti-LZTFL1, anti-γ subunit of AP-1, and anti-α subunit of AP-2 antibodies **(right)**. **(H, left)** Lysate from HEK293FT cells transiently expressing FLAG or LZTFL1-FLAG was analyzed by western blotting using anti-FLAG, anti-β1 subunit and anti-γ subunit of AP-1, and anti-β-actin antibodies or **(I, left)** anti-FLAG, anti-α subunit of AP-2, and anti-actin antibodies. **(H, right)** Cell lysate was immunoprecipitated with anti-FLAG M2 magnetic beads. The LZTFL1-interacting proteins were eluted by 3X FLAG peptide and analyzed by western blotting using anti-FLAG, anti-β1 subunit of AP-1, and anti-γ subunit of AP-1 antibodies or **(I, right)** anti-FLAG and anti-α subunit of AP-2 antibodies.

Analysis of putative protein interaction domains in LZTFL1 (http://elm.eu.org) [27] revealed that LZTFL1 has a DxxFxxLxxxR motif and a dileucine-based motif, ExxxLL, that are known to be important binding sites for clathrin adaptor protein complexes [16]. Conserved motif DxxFxx[L/F]xxxR (hereafter referred to as the DFLR motif) was found in proteins that bind to the appendage region of the β2 subunit of AP-2 [16, 28], while the dileucine-based motif was shown as the binding site for γ and σ1 of AP-1, α and σ2 of AP-2, and δ and σ3 of AP-3 [28]. The conservation of DFLR and dileucine motifs in the orthologs of many vertebrate LZTFL1 (Fig 1A and S1 Fig) strongly suggests a role for these domains in adapter protein complex interactions.

Proteins with the DFLR motif has been shown to bind selectively to the platform subdomain of the β2 subunit of AP-2 [16]. The amino acid sequence alignment of the β1 subunit of AP-1 and the β2 subunit of AP-2 revealed that the subunits share 84% identity and 94% similarity. The amino acid sequences from 834–937 of the β2 subunit of AP-2—which are reported to be the platform to bind the DFLR motif—share 78% identity with the β1 subunit of AP-1, while amino acid residues R834, F837, L838, W841, I876, R879, and Y888—which make bonds with the DFLR motif—share 87% identity (S2 Fig). The DFLR-motif-containing proteins, such as β-arrestin and epsin 1, also bind to the platform subdomain of the β1 subunit of AP-1 [16], suggesting that LZTFL1 could interact with the β1 and β2 subunits of AP-1 and AP-2, respectively.

### LZTFL1 directly interacts with the β subunit of AP-1 and AP-2

To confirm the interaction of LZTFL1 and AP-1, an *in vitro* pull-down assay was carried out with purified MBP-fused LZTFL1 and GST-fused AP-1 subunits (Fig 1B). Results showed that MBP-LZTFL1 directly binds to the β1 subunit of AP-1 (Fig 1C and S3 Fig) but not to the γ, μ1, and σ1 subunits of AP-1. An examination of *in vitro* binding of purified LZTFL1 and its mutant lacking the DFLR domain (ΔNT) or with point mutations in DFLR domain (Fig 1D) showed that neither of these mutants pulled down β1 subunit of AP-1 (Fig 1E) and β2 subunit of AP-2 (Fig 1F). These results indicate that the DFLR domain is critical for binding of LZTFL1 to the β1 subunit of AP-1 and the β2 subunit of AP-2.

To determine the interaction of endogenous LZTFL1, brain lysate from wild-type and *Lztfl1*-knockout mice was immunoprecipitated with anti-LZTFL1-conjugated protein A/G magnetic beads and blotted with antibodies against the γ subunit of AP-1 and the α subunit of AP-2. As expected, the γ subunit of AP-1 and the α subunit of AP-2 were co-immunoprecipitated with LZTFL1 (Fig 1G). To confirm the interaction of LZTFL1 and AP-1, either FLAG or FLAG-tagged LZTFL1 was expressed in HEK293FT cells by transfection with corresponding plasmids and was immunoprecipitated using the anti-FLAG M2 magnetic beads. Western blot analysis of the proteins co-immunoprecipitated with LZTFL1 revealed the presence of the γ and β1 subunits of AP-1, indicating that LZTFL1 interacts with AP-1 (Fig 1H). Similarly, western blot analysis showed that AP-2 was co-immunoprecipitated with LZTFL1 (Fig 1I). These co-immunoprecipitation results indicate that LZTFL1 interacts with AP-1 and AP-2.

### The DFLR motif of LZTFL1 is critical for binding to AP-1 and AP-2

**Fig 2:**
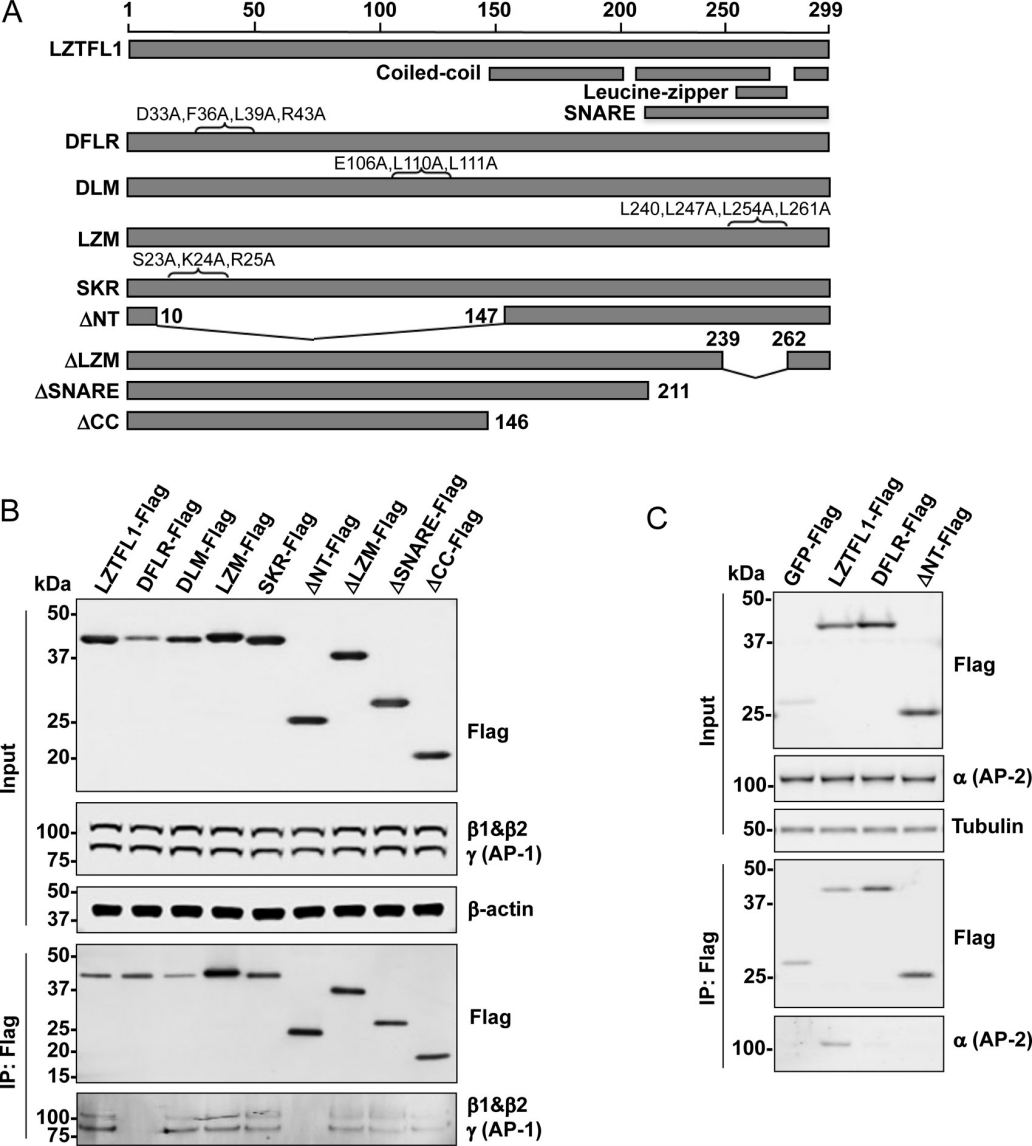
DFLR (DxxFxxLxxxR) motif of LZTFL1 is essential for binding to AP-1 and AP-2 complexes. **(A)** Schematic representation of wild-type and LZTFL1 mutants. **(B)** Cell lysate from HEK293FT cells expressing FLAG-tagged wild-type LZTFL1 or the indicated mutants was analyzed by western blotting using anti-FLAG, anti-γ subunit of AP-1, anti-β1 subunit of AP-1, and anti-β-actin antibodies. Cell lysate was immunoprecipitated with anti-FLAG M2 magnetic beads. The interacting proteins were eluted by 3X FLAG peptide and analyzed by western blotting using anti-FLAG, anti-γ subunit of AP-1, and anti-β1 subunit of AP-1 antibodies. **(C)** Lysate from HEK293FT cells transiently expressing GFP-FLAG, LZTFL1-FLAG, or the indicated mutants was analyzed by western blotting using anti-FLAG, anti-α subunit of AP-2, and anti-tubulin antibodies. Lysate was immunoprecipitated and analyzed by western blotting using anti-FLAG and anti-α subunit of AP-2 antibodies.

LZTFL1 has at least two potential sites that could interact with AP-1. To document whether either of these or any other domains of LZTFL1 are involved in the interaction with AP-1, the following LZTFL1 mutants were tested in a pull-down assay: DFLR motif DxxFxxLxxxR (DFLR mutant with D33A, F36A, L39A, and R43A), dileucine-based motif ExxxLL (DLM mutant with E106A, L110A, and L111A), leucine zipper motif (L)x_6_(L)x_6_(L)x_6_(L) (LZM mutant with L240A, L247A, L254A, and L261A), SKR (highly conserved basic amino acids in the N-terminus, which regulate BBS9 ciliary localization [1], with S23A, K24A, and R25A), N-terminus deletion (ΔNT; amino acids 11–146 were deleted), leucine zipper motif deletion (ΔLZM; amino acids 1–239 were fused with amino acids 262–299), SNARE domain deletion (ΔSNARE; amino acids 212–299 were deleted), and coiled-coil domain deletion (ΔCC; amino acids 147–299 were deleted) (Fig 2A and S2 Table).

Each of these mutants fused with FLAG tag was transiently expressed in HEK293FT cells, and the anti-FLAG tag immunoprecipitates were tested for endogenous AP-1 proteins. While DLM, LZM, SKR, ΔLZM, ΔSNARE, and ΔCC mutants co-immunoprecipitated AP-1, ΔNT and the DFLR mutant did not (Fig 2B). These results demonstrate that the DxxFxxLxxxR motif of LZTFL1 is critical for the interaction of LZTFL1 and AP-1, while the dileucine-based motif (ExxxLL), leucine zipper, SNARE, and SKR domains are not. Similarly, pull-down studies with HEK293FT cells expressing either FLAG-tagged GFP, LZTFL1, or mutants revealed that the DFLR motif of LZTFL1 is crucial for interaction with AP-2 (Fig 2C). Together, these results demonstrate that LZTFL1 interacts with both AP-1 and AP-2 with DFLR motif.

**Fig 3:**
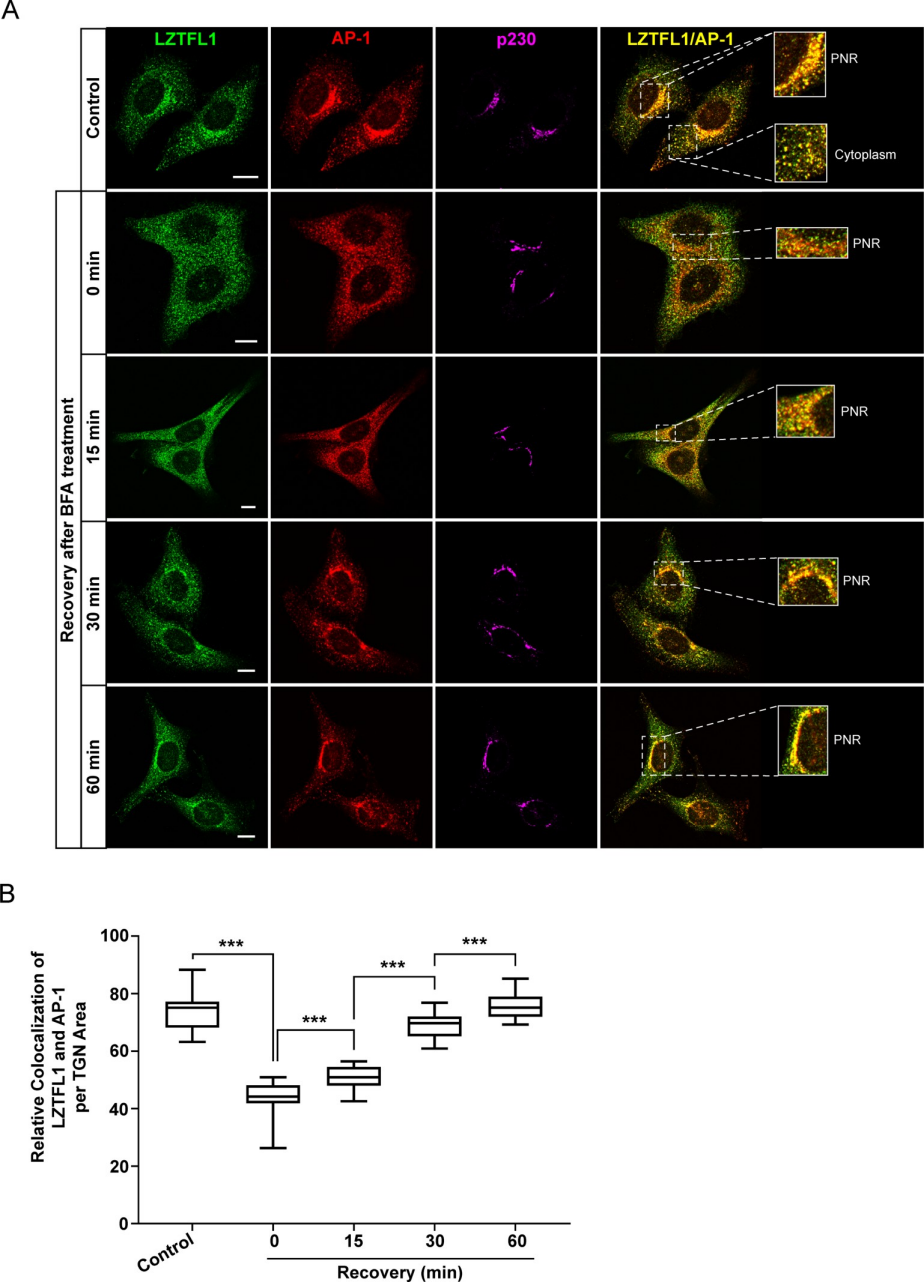
LZTFL1 and AP-1 colocalize at the TGN. **(A)** HeLa cells were incubated with or without 7.5 μg/ml BFA for 2 minutes, washed with complete DMEM to remove BFA, and incubated with complete DMEM for 0, 15, 30, and 60 minutes. Cells were fixed and stained with antibodies to LZTFL1 (green), γ subunit of AP-1 (red) and p230 (TGN marker, magenta). Scale bar = 10 μm. **(B)** Quantitative analysis of the colocalization of LZTFL1 and AP-1 at the TGN (mean + SD). Student’s t-test, n>30 cells, ***p<0.001.

*In vivo* interaction of endogenous LZTFL1 and AP-1 was examined by the immunofluorescence analysis of endogenous LZTFL1 and AP-1. As expected, LZTFL1 colocalized with AP-1 (Fig 3A). Pearson’s correlation coefficient of the colocalization of LZTFL1–AP-1 was 0.74 ± 0.05 (S4 Fig). LZTFL1 antibody used in immunofluorescence did not stain the *LZTFL1*-knockout HeLa cells, demonstrating the specificity of this antibody (S5 Fig).

### AP-1 dependent localization of LZTFL1 at perinuclear region

AP-1 plays important roles in the trafficking of cargo proteins from the TGN to the endosomes, plasma membrane, and immune synapse [9, 29, 30]. ADP-ribosylation factor 1 (Arf1) has been shown to recruit several coat complexes, including AP-1 [31, 32]. Brefeldin A (BFA), a fungal metabolite that is known to inhibit guanine nucleotide exchange factors for Arf1, inactivate AP-1 and disturb the movement of newly synthesized proteins [33–36]. A short term (2 minute) treatment with BFA is known to rapidly and reversibly inhibit the Arf1-dependent recruitment of adaptins (AP-1, AP-2, AP-3 and AP-4) and has been successfully employed to study protein recruitment [33, 34, 37–39]. HeLa cells treated with BFA for 2 minutes were either fixed or the drug was washed out, and the cells were allowed to recover for the times indicated, after which they were fixed and stained for LZTFL1, AP-1, and TGN marker p230. As shown in Fig 3A, LZTFL1 colocalized with AP-1 in the perinuclear region (PNR) encompassing TGN and in the peripheral endosomal compartments. Quantitative analysis of the colocalization of LZTFL1 and AP-1 in the PNR (Fig 3B), showed LZTFL1 and AP-1 were significantly reduced in the PNR. Within 15 minutes after BFA washout, both LZTFL1 and AP-1 were visualized together in the PNR. The level of colocalization of LZTFL1 and AP-1 was increased by 30 minutes of recovery from BFA treatment. The colocalization of LZFTL1 and AP-1 in the PNR reached the steady-state level by 60 minutes after BFA washout. These results potentially indicate that LZTFL1 colocalizes with AP-1 in the PNR in Arf-dependent manner.

**Fig 4:**
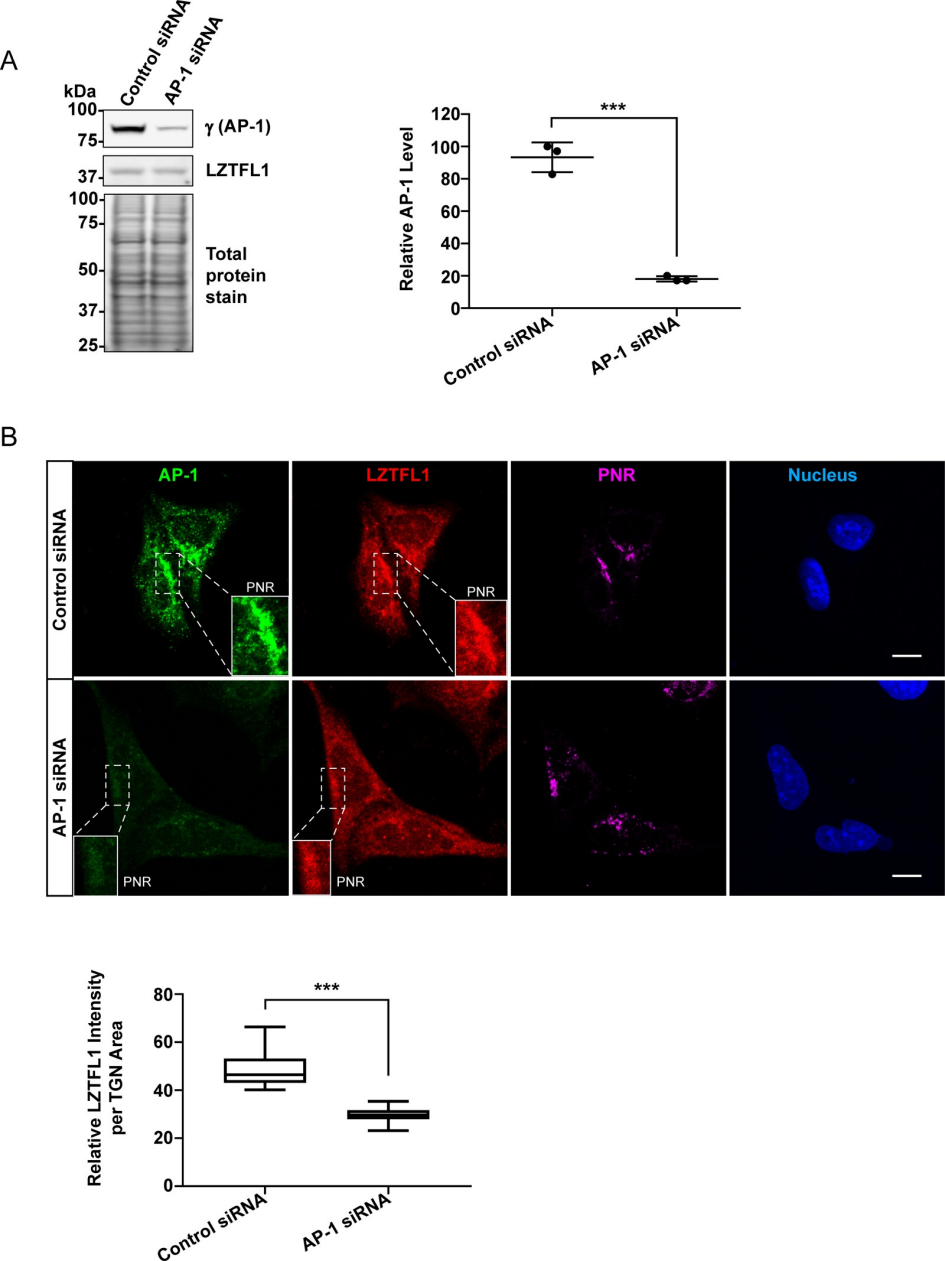
Knockdown of AP-1 reduces the LZTFL1 level at the TGN. **(A)** Cell lysate from control siRNA and AP-1 siRNA treated HeLa cells was analyzed by western blotting using anti-γ subunit of AP-1, anti-LZTFL1 and REVERT staining for total protein (**left**). Quantitation of AP-1 level in control siRNA and AP-1 siRNA treated HeLa cells (mean ± SD) is shown. Student’s t-test, n = 3, ***p<0.001 (**right**). **(B)** Immunofluorescence microscopy of control siRNA and AP-1 siRNA treated HeLa cells fixed and stained with antibodies to γ subunit of AP-1 (green), LZTFL1 (red), p230 (TGN marker, magenta), and DAPI (blue) **(top)**. Scale bar = 10 μm. Quantitative analysis of relative LZTFL1 intensity at the TGN of control siRNA or AP-1 siRNA treated HeLa cells (mean ± SD) is shown **(bottom)**. Student’s t-test, n>120 cells, ***p<0.0001.

To evaluate the role of AP-1 in the localization of LZTFL1 in the PNR, siRNA was used to knockdown AP-1. The AP-1 level in AP-1 siRNA-treated cells was significantly (p<0.001) lower, approximately 70%-80%, than that of control siRNA treated cells (Fig 4A). Under this reduced level of AP-1, total cellular LZTFL1 level was not affected (Fig 4A) but the amount of LZTFL1 in the PNR was significantly (p<0.0001) lower than that of control, indicating that AP-1 is critical for the PNR localization of LZTFL1 (Fig 4B). Together with BFA treatment data, these results indicate that AP-1 plays a role in the trafficking of LZTFL1 to the PNR.

### LZTFL1 interacts with TfR1-bound AP-1

Since AP-1 and AP-2 are involved in trafficking of cargoes, we explored whether LZTFL1 is involved in the transport of plasma membrane receptors. TfR1 is one of the best-characterized cell surface receptors, it is expressed in most of the cells, and both AP-1 and AP-2 are involved in its homeostasis [17–20]. Since it is known that AP-1 is involved in TfR1 transport from the TGN to the endosomes and that AP-2 is critical for the endocytosis of TfR1, we evaluated whether LZTFL1 interacts with TfR1.

**Fig 5:**
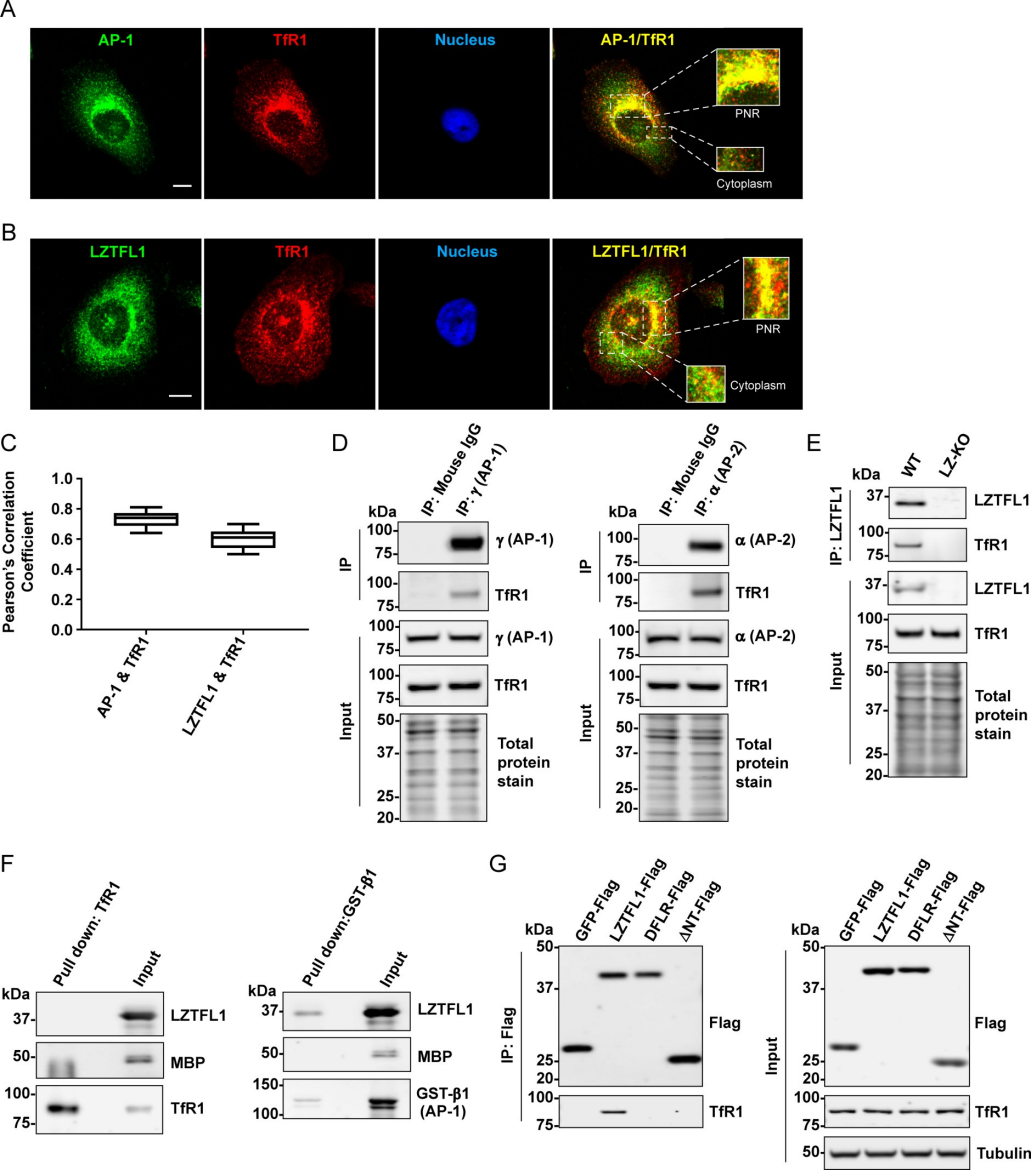
LZTFL1 interacts and colocalizes with TfR1. Immunofluorescence microscopy of HeLa cells fixed and stained with antibodies to **(A)** AP-1 (green), TfR1 (red) and DAPI (blue), scale bar = 10 μm. **(B)** LZTFL1 (green), TfR1 (red) and DAPI (blue), scale bar = 10 μm. **(C)** Pearson’s correlation coefficient of the colocalization of AP-1 or LZTFL1 with TfR1 at the TGN (mean ± SD). n>20 cells from three independent experiments. Costes significance test, p = 1. **(D)** Lysate from HEK293FT cells was immunoprecipitated with mouse IgG and either anti-γ subunit of AP-1 **(left)** or anti-α subunit of AP-2 **(right)** conjugated beads. The interacting proteins were eluted by NuPAGE LDS buffer and analyzed by western blotting using anti- γ subunit of AP-1, anti-α subunit of AP-2, anti-TfR1 and REVERT staining for total protein. **(E)** Lysate from wild-type and *LZTFL1*-knockout HeLa cells was immunoprecipitated with anti-LZTFL1-conjugated beads. The LZTFL1-interacting proteins were eluted by NuPAGE LDS buffer and analyzed by western blotting using anti-LZTFL1, anti-TfR1 and REVERT staining for total protein. **(F)** Factor Xa cleaved MBP-LZTFL1 was incubated with TfR1-Myc-Flag and immunoprecipitated with anti-TfR1 conjugated protein A/G magnetic beads **(left)** or incubated with purified GST-fused β1 subunits of the AP-1 complex and pulled down with Glutathione Sepharose 4B **(right)**. The pulled down proteins were analyzed by western blotting using anti-LZTFL1, anti-MBP, anti-TfR1 and anti-GST antibodies. **(G)** Lysate from HEK293FT cells transiently expressing GFP-FLAG, LZTFL1-FLAG, or the indicated mutants was immunoprecipitated and analyzed by western blotting using anti-FLAG and anti-TfR1 antibodies **(left)**. Lysate was analyzed by western blotting using anti-FLAG, anti-TfR1, and anti-tubulin antibodies **(right)**.

The endogenous LZTFL1, AP-1, and TfR1 in HeLa cells were immunofluorescence-stained with antibodies and examined for colocalization. As shown in Fig 5A, TfR1 colocalized with AP-1 in the PNR and the cytoplasm. This is consistent with the previously reported PNR of TfR1 [17–20, 40–42]. Interestingly, TfR1 also colocalized with LZTFL1 in the PNR and the cytoplasm in a pattern similar to that of AP-1 (Fig 5B). Pearson’s correlation coefficient values for AP-1–TfR1 and LZTFL1–TfR1 colocalization were 0.73 ± 0.05 and 0.60 ± 0.07, respectively (Fig 5C). These results indicate the potential interaction of LZTFL1 with a protein complex containing AP-1 and TfR1.

To further evaluate the interaction of TfR1 with AP-1 or AP-2, HEK293FT cell lysate was immunoprecipitated with anti-γ subunit of AP-1 and anti-α subunit of AP-2 conjugated protein A/G magnetic beads and blotted with antibodies against TfR1 (Fig 5D). As expected, TfR1 was co-immunoprecipitated with γ subunit of AP-1 and α subunit of AP-2, indicating that TfR1 interacts with γ subunit of AP-1 and α subunit of AP-2 *in vitro*.

To confirm the interaction of LZTFL1 and TfR1, lysate from wild-type and *LZTFL1*-knockout HeLa cells was immunoprecipitated with anti-LZTFL1-conjugated protein A/G magnetic beads and blotted with antibodies against TfR1 (Fig 5E). As expected, TfR1 was co-immunoprecipitated with LZTFL1, indicating that LZTFL1 interacts with TfR1 *in vitro*. To determine whether LZTFL1 directly binds to TfR1, an *in vitro* pull-down assay was carried out with purified MBP-fused LZTFL1 cleaved by Factor Xa and either TfR1-Myc-Flag or GST-fused β1 subunit of AP-1 (as a positive control) (Fig 5F). Pull down results showed that *in vitro* LZTFL1 directly binds to β1 subunit of AP-1 but under the same conditions, LZTFL1 does not bind to TfR1. These results suggest that LZTFL1 does not interact directly with TfR1 but indirectly through AP-1 or AP-2.

Next, we examined whether the DxxFxxLxxxR motif of LZTFL1 is critical for the indirect binding to TfR1 through AP-1. Coimmunoprecipitation of lysates from HEK293FT cells expressing either FLAG-tagged GFP, wild-type LZTFL1, ΔNT and DFLR mutants revealed that the DxxFxxLxxxR motif of LZTFL1 is critical for the interaction of LZTFL1 and TfR1 (Fig 5G). These data support the concept that LZTFL1 interacts with TfR1-bound AP-1.

### LZTFL1 regulates the trafficking of TfR1 to the cell surface

**Fig 6:**
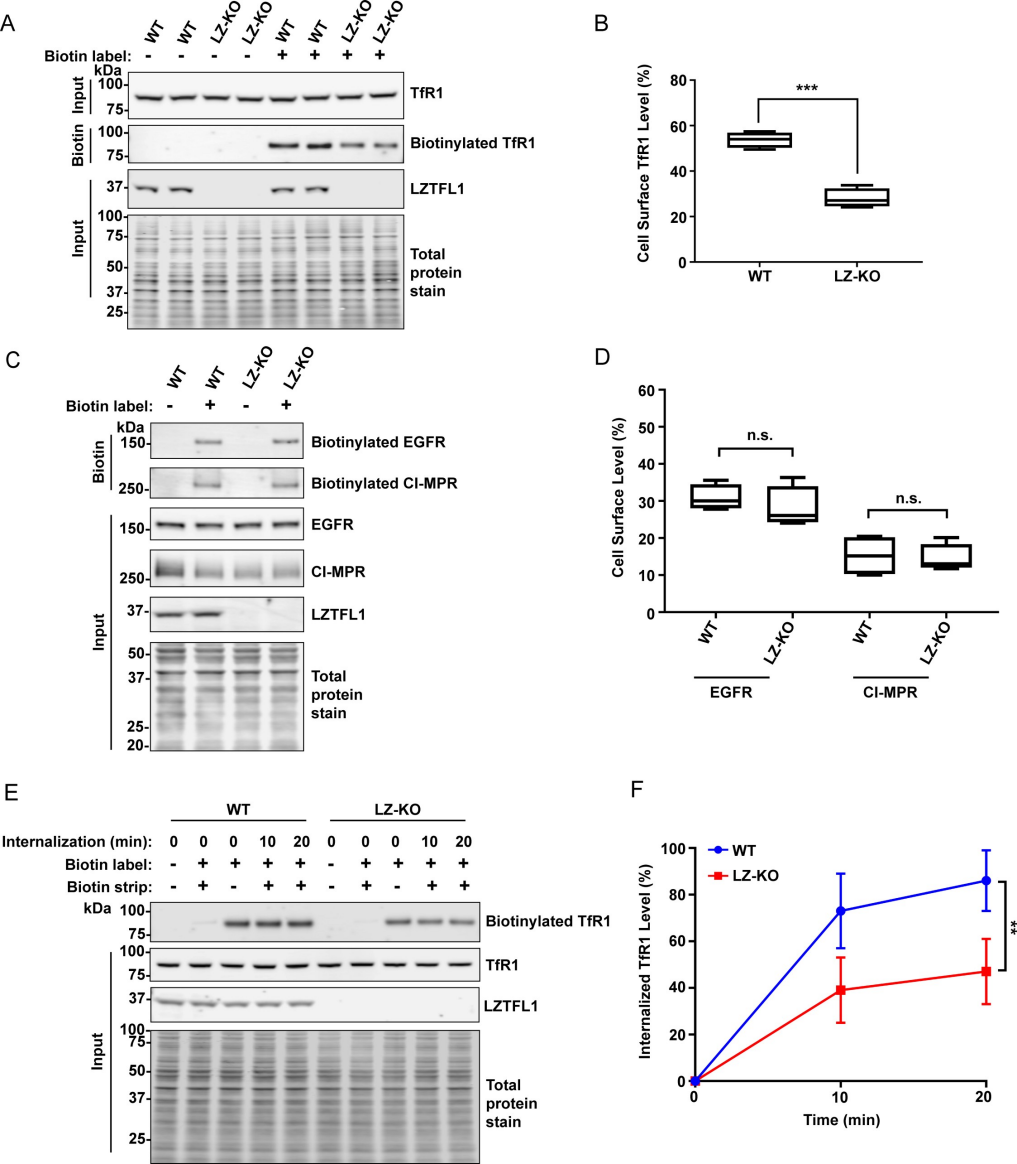
LZTFL1 regulates cell surface level and internalization rate of TfR1. **(A)** Wild-type and *LZTFL1*-knockout HeLa cells were labeled with 2 mM Sulfo-NHS-LC-Biotin. Biotin-labeled proteins were extracted as described in the Methods and were analyzed by western blotting using anti-TfR1, anti-LZTFL1, and REVERT staining for total protein. **(B)** Quantitation of cell surface TfR1 level in wild-type and *LZTFL1*-knockout HeLa cells (mean ± SD). Student’s t-test, n = 3, ***p<0.0001. **(C)** Biotin-labeled proteins were extracted from wild-type and *LZTFL1*-knockout HeLa cells as described in the Methods and were analyzed by western blotting using anti-EGFR, anti-CI-MPR, anti-LZTFL1, and REVERT staining for total protein. **(D)** Quantitation of cell surface EGFR and CI-MPR levels in wild-type and LZTFL1-knockout HeLa cells (mean + SD). Student’s t-test, n = 4, n.s., not significant. **(E)** Wild-type and *LZTFL1*-knockout HeLa cells were labeled with 2 mM Sulfo-NHS-SS-Biotin at 4°C. Biotin-labeled cells were incubated with complete DMEM for 0, 10, and 20 minutes to allow protein internalization. The remaining biotin-labeled cell surface proteins were removed with cold 100 mM Mesna followed by quenching with 120 mM iodoacetamide. Biotin-labeled proteins were then extracted as described in the Methods and were analyzed by western blotting using anti-TfR1 antibody, anti-LZTFL1 antibody, and REVERT staining for total protein. **(F)** Quantitation of internalized TfR1 level in wild-type and *LZTFL1*-knockout HeLa cells (mean ± SD). Student’s t-test, n = 4, **p<0.005.

Next, to understand the role of LZTFL1 in TfR1 localization and endocytosis, the cell surface level of TfR1 was estimated in wild-type and *LZTFL1*-knockout HeLa cells by biotinylation of cell surface proteins. Wild-type and *LZTFL1*-knockout cells had similar levels of total TfR1 (Fig 6A), AP-1 and AP-2 (S7 Fig). However, the cell surface level of TfR1 in *LZTFL1*-knockout cells was significantly (p<0.0001) lower, about 40%-50%, than that of wild-type cells (Fig 6A and 6B), suggesting LZTFL1 has a role in the homeostasis of TfR1. Next, we examined whether LZTFL1 regulates the cell surface level of some other receptors known to be trafficked by AP-1 complex. Epidermal growth factor receptor (EGFR) and cation-independent mannose-6-phosphate receptor (CI-MPR) are known to be trafficked to the cell surface by AP-1 complex [43–49]. Estimation of cell surface proteins by biotinylation analysis revealed that wild-type and *LZTFL1*-knockout cells had similar levels of total and cell surface levels of EGFR and CI-MPR (Fig 6C and 6D). These results indicate that LZTFL1 specifically regulates the cell surface level of TfR1.

**Fig 7:**
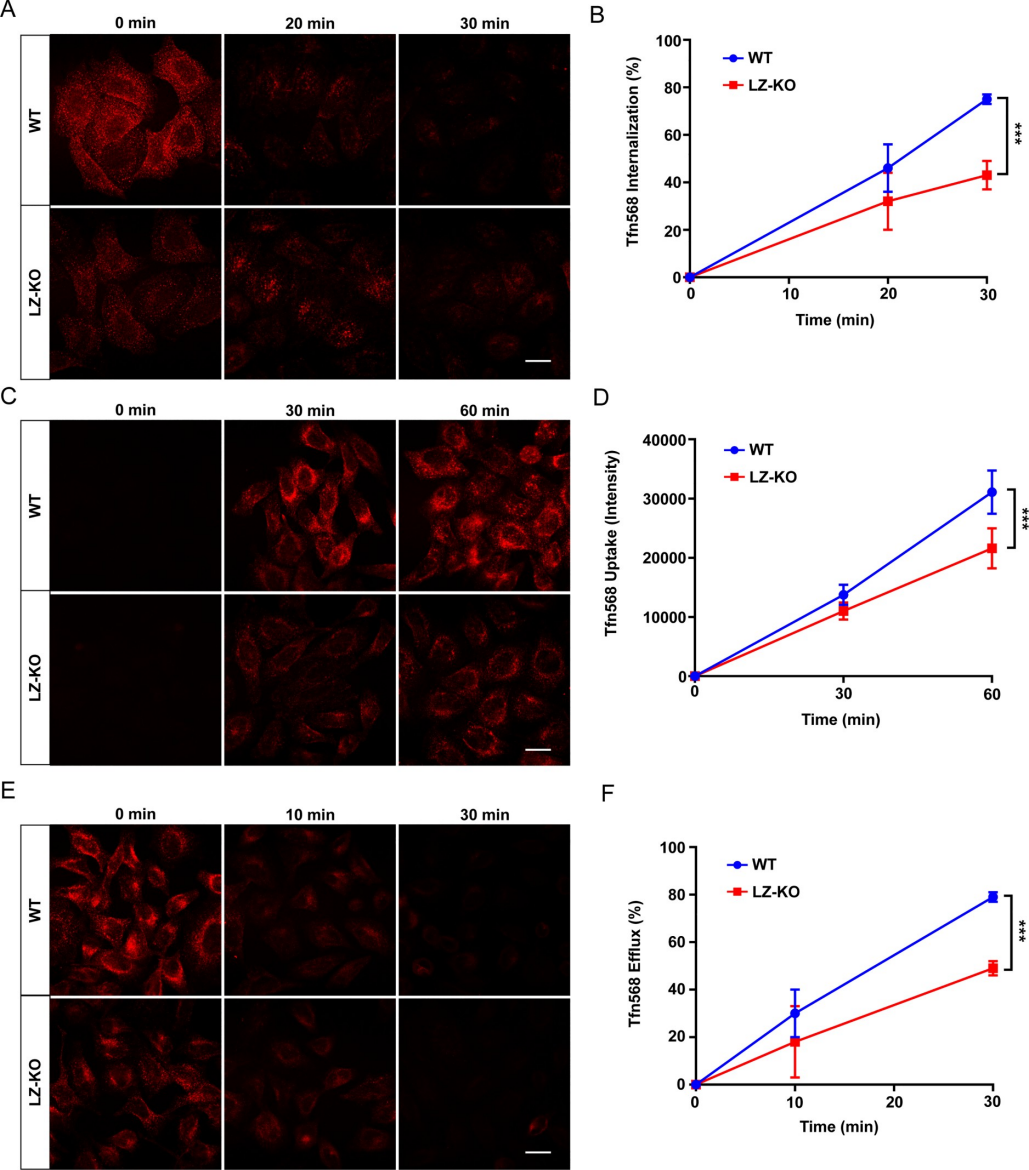
LZTFL1 regulates transferrin internalization, uptake, and efflux. **(A)** Wild-type and *LZTFL1*-knockout HeLa cells were loaded with 25 μg/ml of Tfn-568 on ice for 30 minutes, washed, and incubated with complete DMEM for 0, 20, and 30 minutes at 37°C. Time course of Tfn-568 internalization was monitored by loss of fluorescence. Scale bar = 20 μm. **(B)** Quantitation of Tfn-568 internalization intensity as a percentage normalized with the intensity at time point 0 (100%) in wild-type and *LZTFL1*-knockout HeLa cells (mean ± SD). Student’s t-test, n>100 cells, ***p<0.0001. **(C)** Wild-type and *LZTFL1*-knockout HeLa cells were incubated with 25 μg/ml Tfn-568 for the indicated times, and the Tfn uptake level was measured by the accumulation of Tfn-568. Scale bar = 20 μm. **(D)** Quantitation of Tfn-568 accumulation in wild-type and *LZTFL1*-knockout HeLa cells (mean ± SD). Student’s t-test, n>100 cells, ***p<0.0001. **(E)** Wild-type and *LZTFL1*-knockout HeLa cells were loaded with 25 μg/ml Tfn-568 for 30 minutes, washed, and incubated with complete DMEM for the indicated times. Time course of Tfn-568 efflux was monitored by loss of fluorescence. Scale bar = 20 μm. **(F)** Quantitation of Tfn-568 efflux intensity as a percentage normalized with the intensity at time point 0 (100%) in wild-type and *LZTFL1*-knockout HeLa cells (mean ± SD). Student’s t-test, n>100 cells, ***p<0.0001.

To understand why the *LZTFL1*-knockout cells had lower TfR1 on the cell surface, the rate of TfR1 internalization was estimated. Wild-type and *LZTFL1*-knockout HeLa cells were labeled with biotin, and the internalization rate of biotin-labeled TfR1 was monitored after incubating the cells at 37°C, stripping the biotin-labeled cell surface proteins with Mesna, and quantitating the cellular level of biotin-labeled TfR1. The internalization of biotin-labeled TfR1 in *LZTFL1*-knockout cells at 10 and 20 minutes was significantly (p<0.005) lower than that of wild-type cells (Fig 6E and 6F), showing that LZTFL1 modulates the rate of TfR1 internalization. This conclusion was supported by the lower rate of transferrin internalization observed in *LZTFL1*-knockout cells than in wild-type cells. The wild-type and *LZTFL1*-knockout cells were incubated with fluorescently labeled transferrin, Tfn-568, at 4°C. The rate of transferrin internalization was monitored by the loss of the Tfn-568 intensity after incubating the cells with complete medium at 37°C for various lengths of time (Fig 7A). By 30 minutes, the difference in transferrin internalization between wild-type and knockout cells was significant (p<0.0001), about 75 ± 2% of Tfn-568 in wild-type compared to 43 ± 6% in knockout cells (Fig 7B). The lower rate of transferrin internalization in *LZTFL1*-knockout cells confirms that LZTFL1 plays a role in TfR1 internalization.

Next, we compared the rate of transferrin uptake by TfR1 between the wild-type and *LZTFL1*-knockout cells by incubating the cells with serum-free medium and then with Tfn-568. As expected, *LZTFL1*-knockout cells showed a significantly (p<0.0001) reduced rate of accumulation and the steady-state level of transferrin, indicating a role for LZTFL1 in TfR1 activity (Fig 7C and 7D).

To evaluate the TfR1 turnover, the rate of transferrin efflux was studied [50]. After loading the cells with Tfn-568 for 30 minutes in serum-free medium, cells were washed and the efflux of Tfn-568 was monitored by incubating the cells with complete medium (Fig 7E). By 30 minutes, wild-type cells exported significantly more Tfn (79 ± 2%) than knockout cells (49 ± 3%) (p<0.0001) (Fig 7F).

In addition to TfR1, TfR2 is also involved in transferrin uptake [51–53]. To rule out any role for TfR2 in the lowered level of transferrin uptake and efflux seen in *LZTFL1-*knockout cells, we examined the binding of LZTFL1 to TfR2. Results shown in S8 Fig indicate that LZTFL1 does not bind to TfR2 under the condition tested where it binds to TfR1. We used immunofluorescence staining to estimate the cell surface level of TfR2 because biotinylation methods could not be used. Results showed that wild-type and *LZTFL1*-knockout cells had similar level of cell surface TfR2 (S9 Fig). These data suggest that TfR2 may not be a major player in the regulation of transferrin uptake and efflux in *LZTFL1*-knockout cells.

Thus, these transferrin uptake, efflux, and internalization data indicate that LZTFL1 plays an important role in the regulation of TfR1 turnover and iron uptake.

## Discussion

Putative protein interaction domain analysis (http://elm.eu.org) revealed that LZTFL1 has a number of known motifs. The SNARE and coiled-coil domains located at the C-terminus of LZTFL1 are known to be involved in membrane fusion [54, 55]. The dileucine-based motif and DFLR (DxxFxxLxxxR) motif at the N-terminus of LZTFL1 could associate with clathrin adaptor protein complexes [16, 28]. Proteins with a dileucine-based motif bind to the combination of the γ and σ1 subunits of AP-1 [28]. However, the co-immunoprecipitation of cell extracts revealed that the mutation of the dileucine-based motif of LZTFL1 did not affect the AP-1 binding, suggesting that the dileucine-based motif of LZTFL1 is not critical for interaction with AP-1.

Several adaptor accessory proteins, such as β-arrestins, autosomal recessive hypercholesterolemia protein, epsin, Eps15, Scy1-like1, and Tom1, bind to the β2-platform subdomain [16, 29, 56–58]. The interaction between the β2 subunit of AP-2 and these accessory proteins is mediated by the consensus DFLR motif, strongly indicating that LZTFL1 is an adaptor accessory protein of AP-1 and AP-2. Based on the interaction of LZTFL1 with the β1 and β2 subunits of AP complexes and the requirement of the DFLR motif for this interaction, we speculate that the DFLR motif in the N-terminus of LZTFL1 binds to the platform subdomain of the β1 and β2 subunits of AP-1 and AP-2, respectively.

AP-1 plays important roles in vesicle trafficking at the TGN and endosomes, and it regulates the TGN–endosomal and the TGN–basolateral plasma membrane transport pathways [59]. The appendages of the large subunits of adaptor protein complexes are targets for regulatory proteins that are involved in various phases of vesicle formation, scission from the plasma membrane, and vesicle uncoating [60]. The PNR contains endosomal vesicles (early, late or recycling endosomes) including clathrin-mediated and clathrin-independent vesicles with cargoes, endoplasmic reticulum, TGN, and lysosomes [61–64]. AP-1 also has been shown to localize in the PNR encompassing TGN [65]. BFA is known to rapidly and reversibly inhibit the Arf-dependent recruitment of adaptors including AP-1 [33, 34, 37–39]. The rapid reduction of colocalized AP-1 and LZTFL1 in the PNR upon the exposure to BFA and the recovery as early as 15 min after BFA removal indicate that both AP-1 and LZTFL1 are BFA sensitive Arf1-dependent adaptors. Further studies are needed to determine whether the LZTFL1 binds AP-1 in the PNR or binds at a different compartment and transported as a complex to the PNR. The colocalization of LZTFL1 and AP-1 in the PNR indicates that LZTFL1 could be part of the AP-1-containing vesicle that traffics from the PNR to target compartments such as the plasma membrane, immune synapse, and cilia. This hypothesis was further supported by the observation that AP-1 was abnormally distributed in photoreceptor cells of *Lztfl1*-knockout mice [7]. Of note, an alternatively spliced mRNA (Genbank Accession: NP_001263308) codes for an isoform of LZTFL1 that lacks the DFLR domain, suggesting the adaptor protein complex independent functions for LZTFL1.

LZTFL1 is an important regulator of ciliary trafficking of BBSome and hedgehog signal transducer, Smoothened [1]. Interestingly, LZTFL1 is not only involved in protein localization to cilia, but it is also shown to bind to membrane receptors such as E-cadherin and insulin receptor [3, 66]. In this study, we have shown for the first time that LZTFL1 binds to AP-1 and AP-2 *in vitro* and may be involved in the trafficking of TfR1.

TfR1 is a membrane receptor that constitutively recycles between the plasma membrane and endosomes [28]. In *LZTFL1*-knockout cells, the total cellular TfR1 level was not affected, but the cell surface TfR1 level was lower while the total as well as cell surface levels of EGFR and CI-MPR were not affected, indicating that LZTFL1 specifically regulates the trafficking of TfR1 to the cell surface. In addition, the rate of TfR1 internalization as well as transferrin uptake and efflux were also lower in *LZTFL1*-knockout cells, indicating that LZTFL1 participates in the TfR1 recycling pathway. Fine-tuned control of actin polymerization has been shown to play an important role in membrane receptor retrieval and recycling [67]. Based on the recent evidence that LZTFL1 binds to actin and actin-binding proteins [68], we speculate that LZTFL1 may affect the actin cytoskeleton and influence the TfR1 recycling.

In summary, we obtained evidence to show that LZTFL1 directly interacts with the β subunits of AP-1 and AP-2 and facilitates the recycling of TfR1.

## Supporting information

S1 Fig Amino acid alignment of LZTFL1 from different species. Amino acid sequences of LZTFL1 from different species are aligned to illustrate the conservation of DFLR and dileucine-based motifs. The red and blue lines under the sequences represent the conserved DFLR and dileucine-based motifs, respectively.
(TIF)

S2 Fig Amino acid alignment of human β1 subunit of AP-1 and β2 subunit of AP-2. The amino acid sequence alignment of human β1 subunit of AP-1 (949 amino acids) and β2 subunit of AP-2 (937 amino acids) was generated using Cell Biology Unit Web Server software (http://xylian.igh.cnrs.fr/bin/align-guess.cgi). The amino acid residues shown in red represent the binding sites for the DFLR motif. Identical amino acids are indicated with a colon (:). Similar amino acids are indicated with a period (.). Dissimilar amino acids are indicated as a blank. Gaps required for optimal alignment are indicated by dashes.
(TIF)

S3 Fig Full immunoblot image of GST pull-down. Complete western blot gel displayed in Fig 1C. Dashed selections indicate cropped samples shown for GST.
(TIF)

S4 Fig Degree of colocalization of LZTFL1 and AP-1. Pearson’s correlation coefficient of the colocalization of LZTFL1 with AP-1 data shown in Fig 3A (mean + SD). Student’s t-test, n >20 cells from three independent experiments, ***p<0.0001. Costes significance test, p = 1.
(TIF)

S5 Fig The specificity of LZTFL1 antibody. Immunofluorescence microscopy of wild-type and *LZTFL1*-knockout HeLa cells fixed and stained with LZTFL1 antibody (red) and DAPI (blue). n>20 cells from two independent experiments. Scale bar = 10 μm.
(TIF)

S6 Fig Degree of colocalization of LZTFL1 and AP-1 at TGN. Pearson’s correlation coefficient of the colocalization of LZTFL1 and AP-1 at TGN of DMSO or BFA treated HeLa cells data shown in Fig 3A (mean + SD). Student’s t-test, n >20 cells from three independent experiments, ***p<0.0001. Costes significance test, p = 1.
(TIF)

S7 Fig Wild-type and *LZTFL1*-knockout HeLa cells had similar levels of AP-1 and AP-2. **(A)** Cell lysate of wild-type and *LZTFL1*-knockout HeLa cells was analyzed by western blotting using anti-γ subunit of AP-1, anti-α subunit of AP-2, anti-LZTFL1, and anti-actin antibodies. Immunofluorescence microscopy was conducted on wild-type and *LZTFL1*-knockout HeLa cells fixed and stained with antibodies to **(B)** γ subunit of AP-1 (red) and DAPI (blue) and **(C)** α subunit of AP-2 (green) and DAPI (blue). n>40 cells from two independent experiments. Scale bar = 20 μm.
(TIF)

S8 Fig LZTFL1 did not interact with TfR2. Cell lysate from HEK293FT cells expressing GFP-FLAG or LZTFL1-FLAG was analyzed by western blotting using anti-FLAG, anti-TfR2 and anti-tubulin antibodies. Cell lysate was immunoprecipitated with anti-FLAG M2 magnetic beads. The interacting proteins were eluted by 3X FLAG peptide and analyzed by western blotting using anti-FLAG and anti-TfR2 antibodies.
(TIF)

S9 Fig Wild-type and *LZTFL1*-knockout HeLa cells had similar cell surface TfR2 level. **(A)** Immunofluorescence microscopy of wild-type and *LZTFL1*-knockout HeLa cells fixed and stained with TfR2 antibody under non-permeabilization condition. n > 40 cells from two independent experiments. Scale bar = 20 μm. **(B)** Quantitation of cell surface TfR2 level in wild-type and *LZTFL1*-knockout HeLa cells (mean ± SD). Student’s t-test, n.s., not significant.
(TIF)

S1 Table LZTFL1-interacting proteins identified by LC-MS/MS. (TIF)

S2 Table The amino acid sequence of LZTFL1 mutants. (TIF)
