# Supplementary figures and images for: Leucine zipper transcription factor-like 1 binds adaptor protein complex-1 and 2 and participates in trafficking of transferrin receptor 1
Source: PLoS One. 2020 Jan 2;15(1):e0226298. doi: 10.1371/journal.pone.0226298 (PMC6939906; doi:10.1371/journal.pone.0226298)

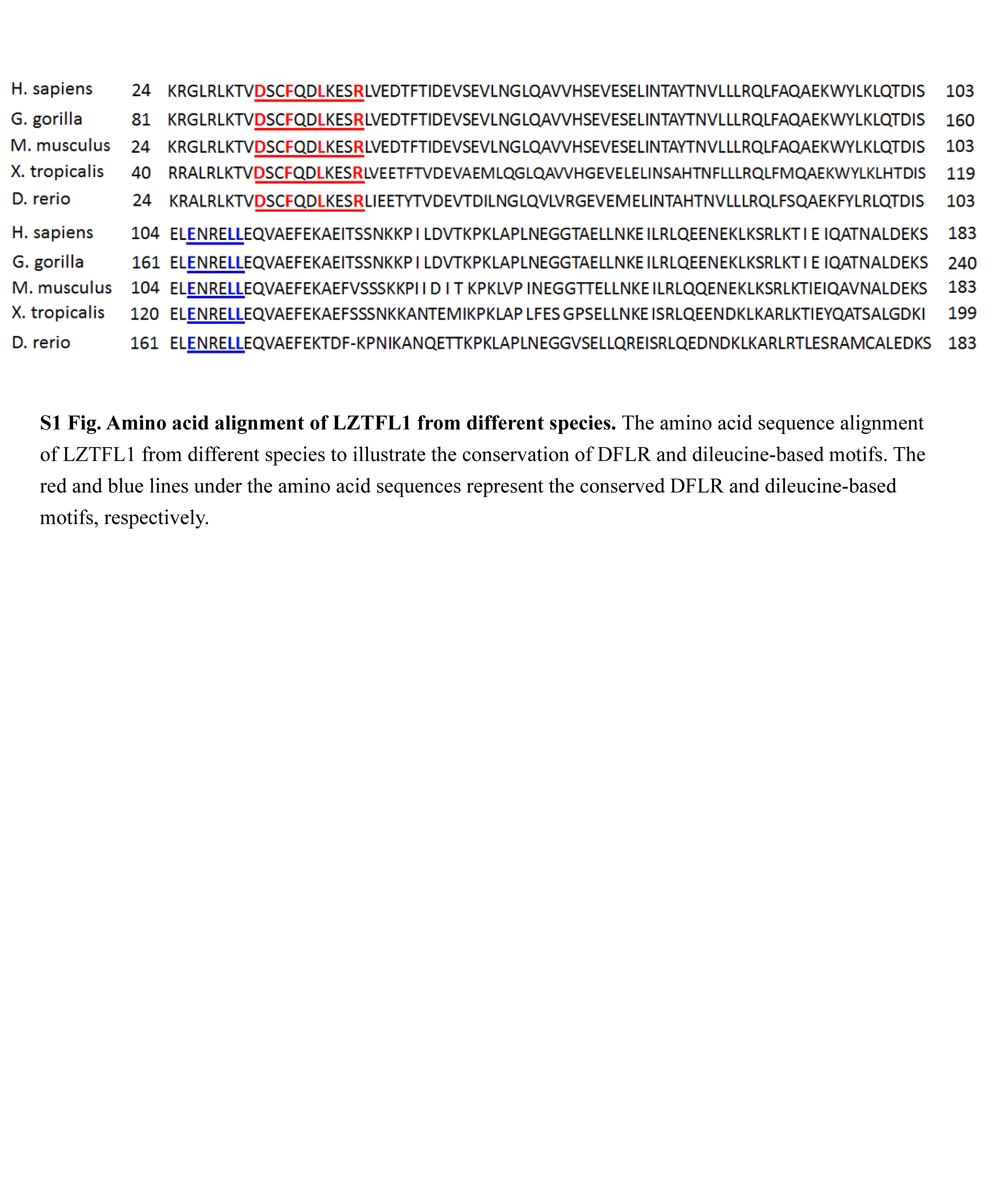

Supplement: S1 Fig — Amino acid sequences of LZTFL1 from different species are aligned to illustrate the conservation of DFLR and dileucine-based motifs. The red and blue lines under the sequences represent the conserved DFLR and dileucine-based motifs, respectively. (TIF) [file pone.0226298.s001.tif]

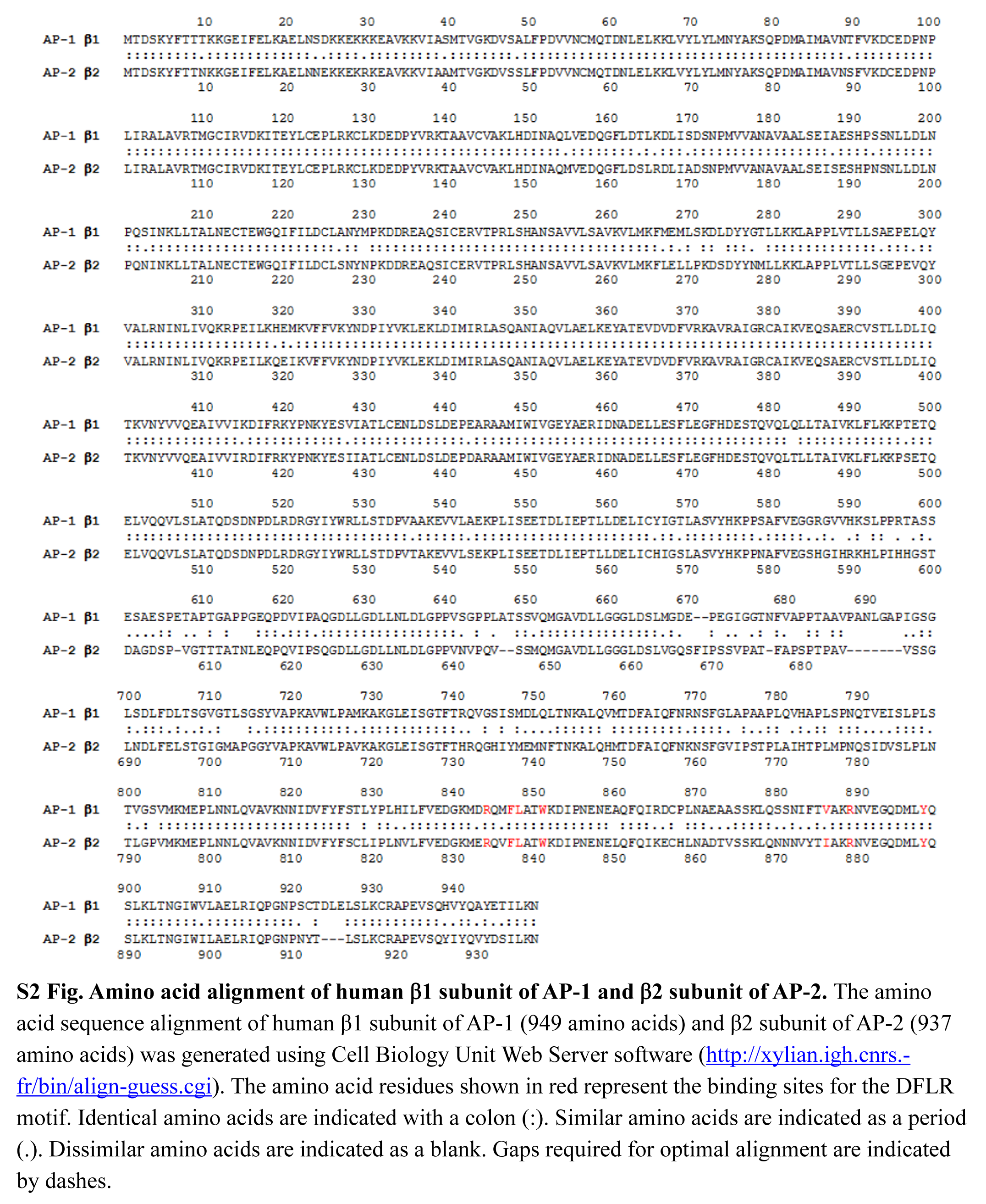

Supplement: S2 Fig — The amino acid sequence alignment of human β1 subunit of AP-1 (949 amino acids) and β2 subunit of AP-2 (937 amino acids) was generated using Cell Biology Unit Web Server software (http://xylian.igh.cnrs.fr/bin/align-guess.cgi). The amino acid residues shown in red represent the binding sites for the DFLR motif. Identical amino acids are indicated with a colon (:). Similar amino acids are indicated with a period (.). Dissimilar amino acids are indicated as a blank. Gaps required for optimal alignment are indicated by dashes. (TIF) [file pone.0226298.s002.tif]

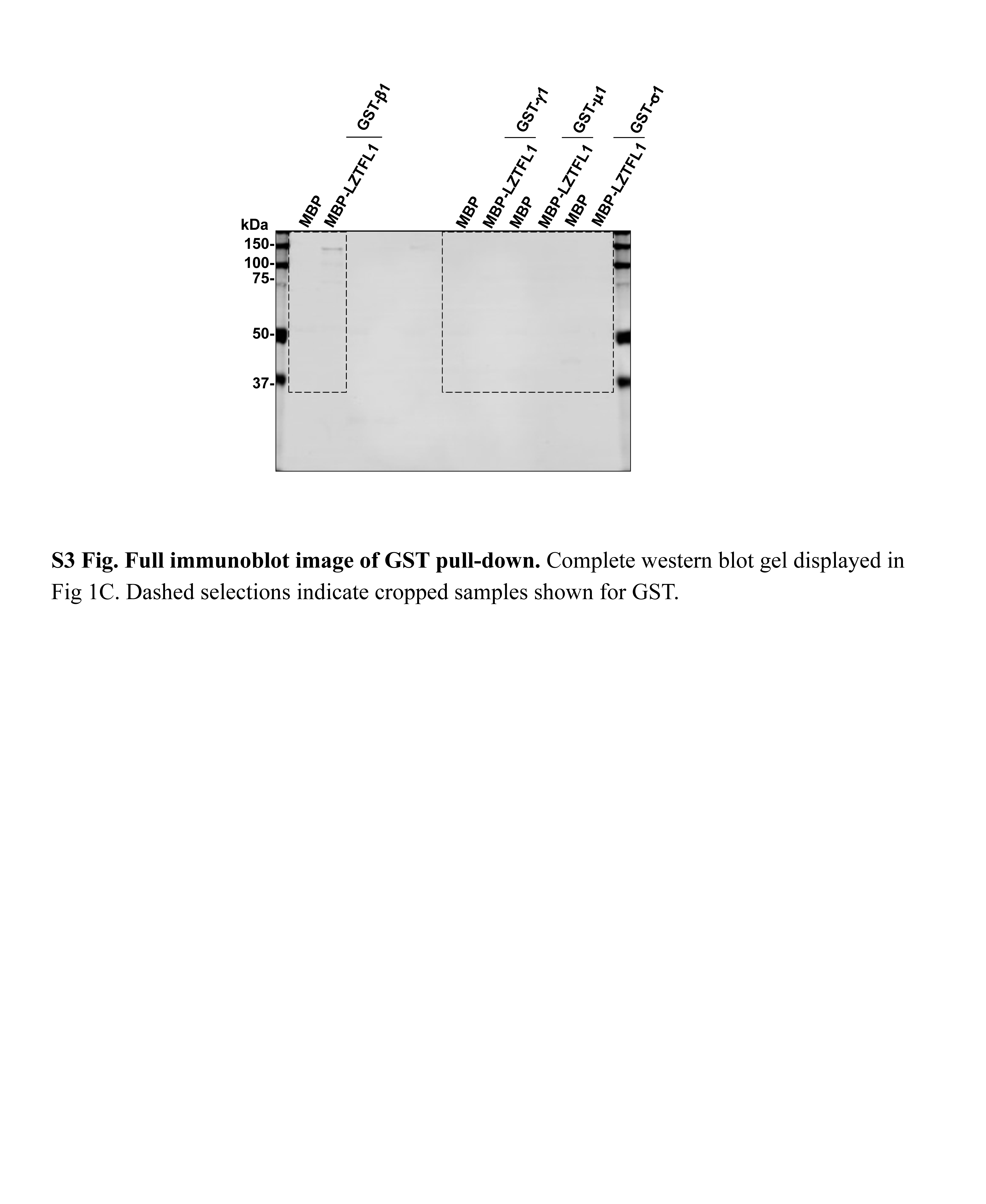

Supplement: S3 Fig — Complete western blot gel displayed in Fig 1C. Dashed selections indicate cropped samples shown for GST. (TIF) [file pone.0226298.s003.tif]

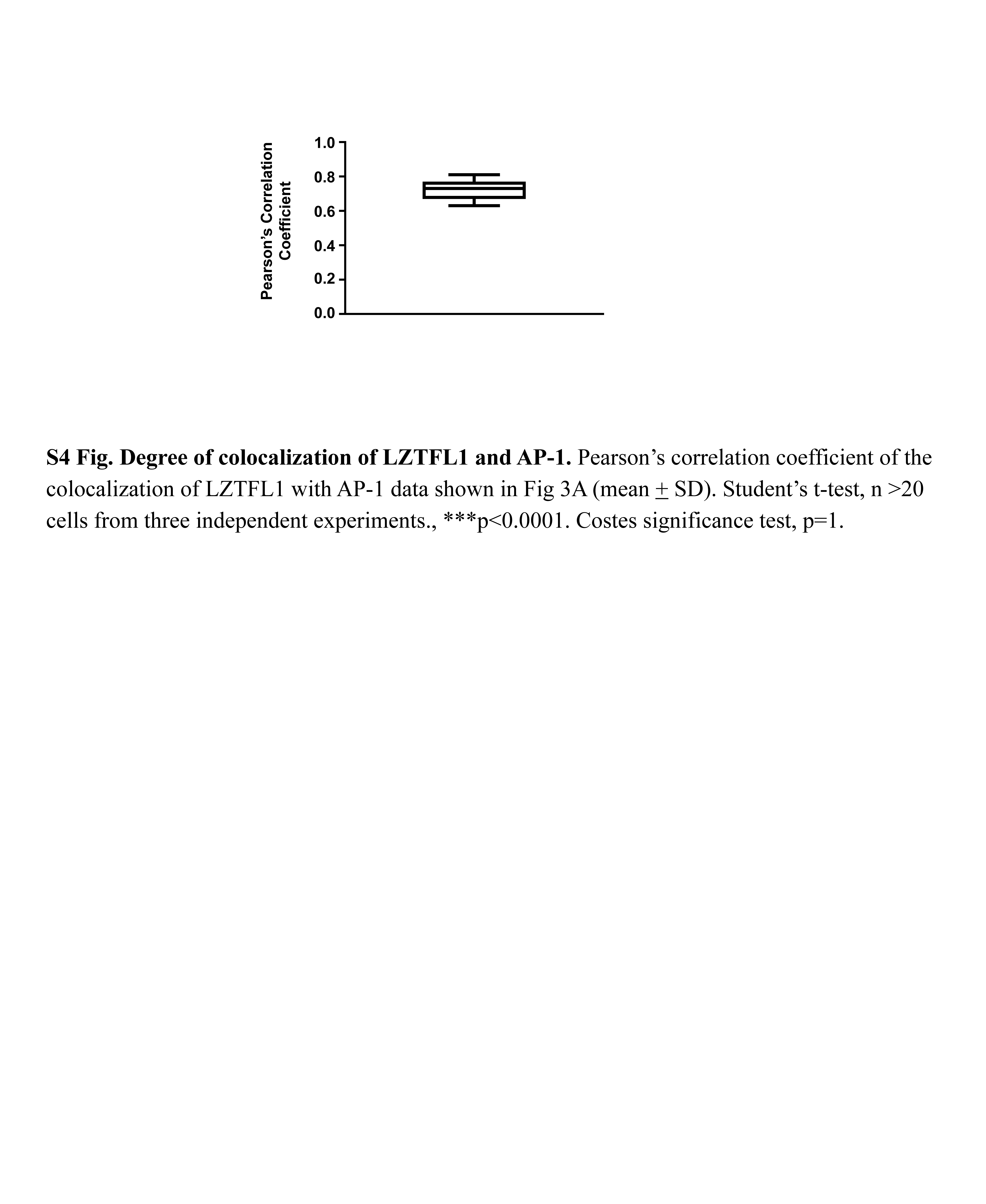

Supplement: S4 Fig — Pearson’s correlation coefficient of the colocalization of LZTFL1 with AP-1 data shown in Fig 3A (mean + SD). Student’s t-test, n >20 cells from three independent experiments, ***p<0.0001. Costes significance test, p = 1. (TIF) [file pone.0226298.s004.tif]

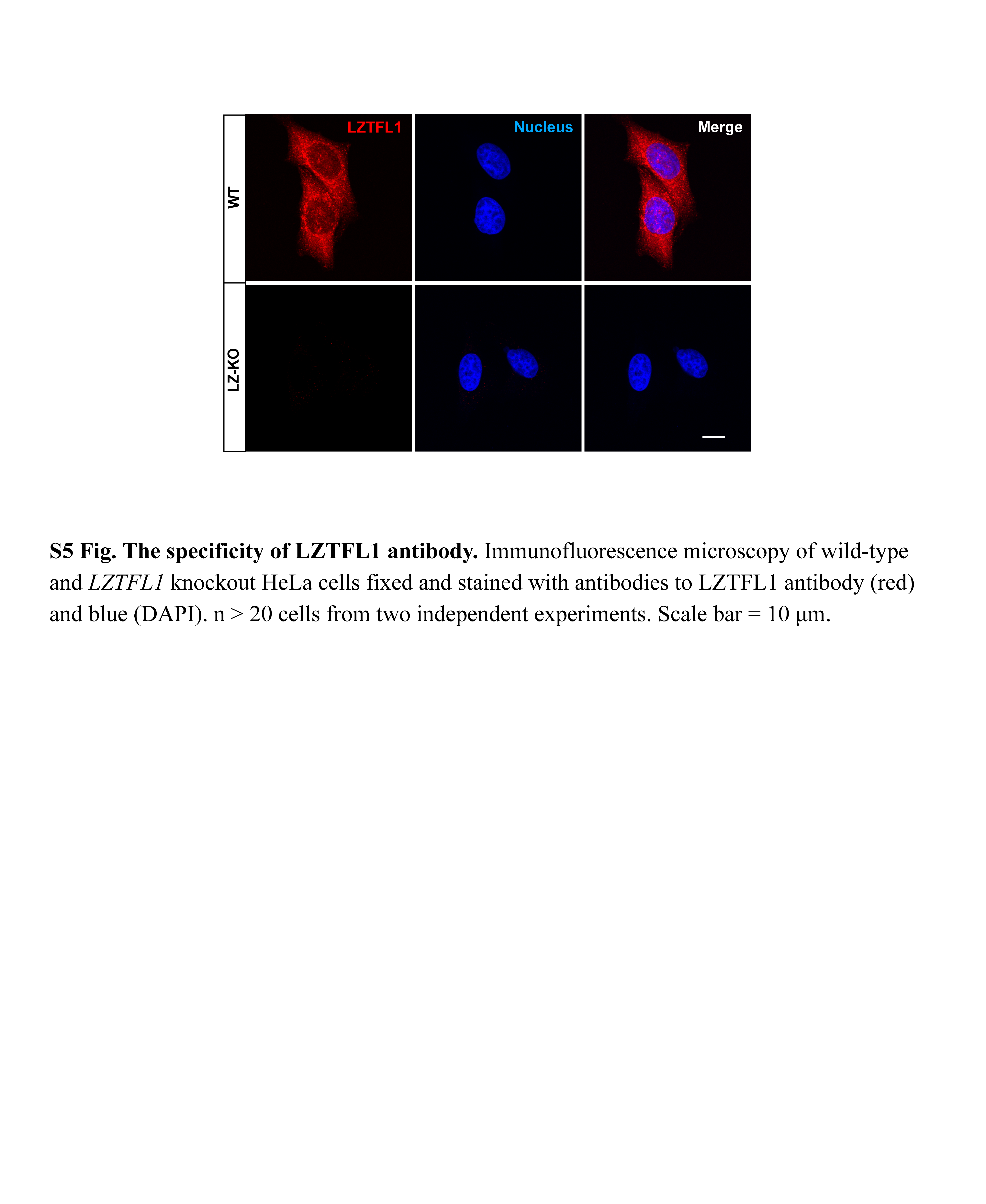

Supplement: S5 Fig — Immunofluorescence microscopy of wild-type and LZTFL1-knockout HeLa cells fixed and stained with LZTFL1 antibody (red) and DAPI (blue). n>20 cells from two independent experiments. Scale bar = 10 μm. (TIF) [file pone.0226298.s005.tif]

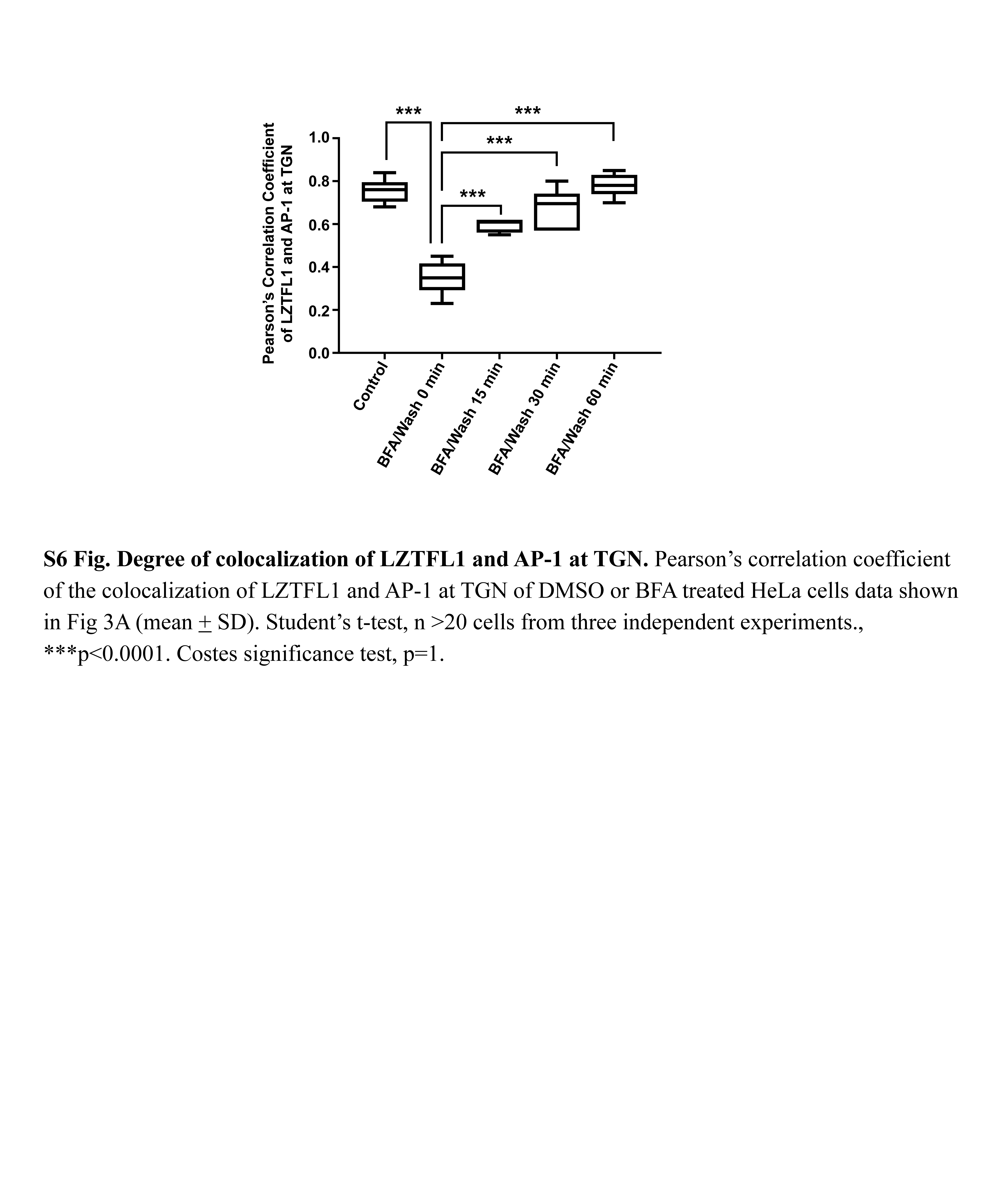

Supplement: S6 Fig — Pearson’s correlation coefficient of the colocalization of LZTFL1 and AP-1 at TGN of DMSO or BFA treated HeLa cells data shown in Fig 3A (mean + SD). Student’s t-test, n >20 cells from three independent experiments, ***p<0.0001. Costes significance test, p = 1. (TIF) [file pone.0226298.s006.tif]

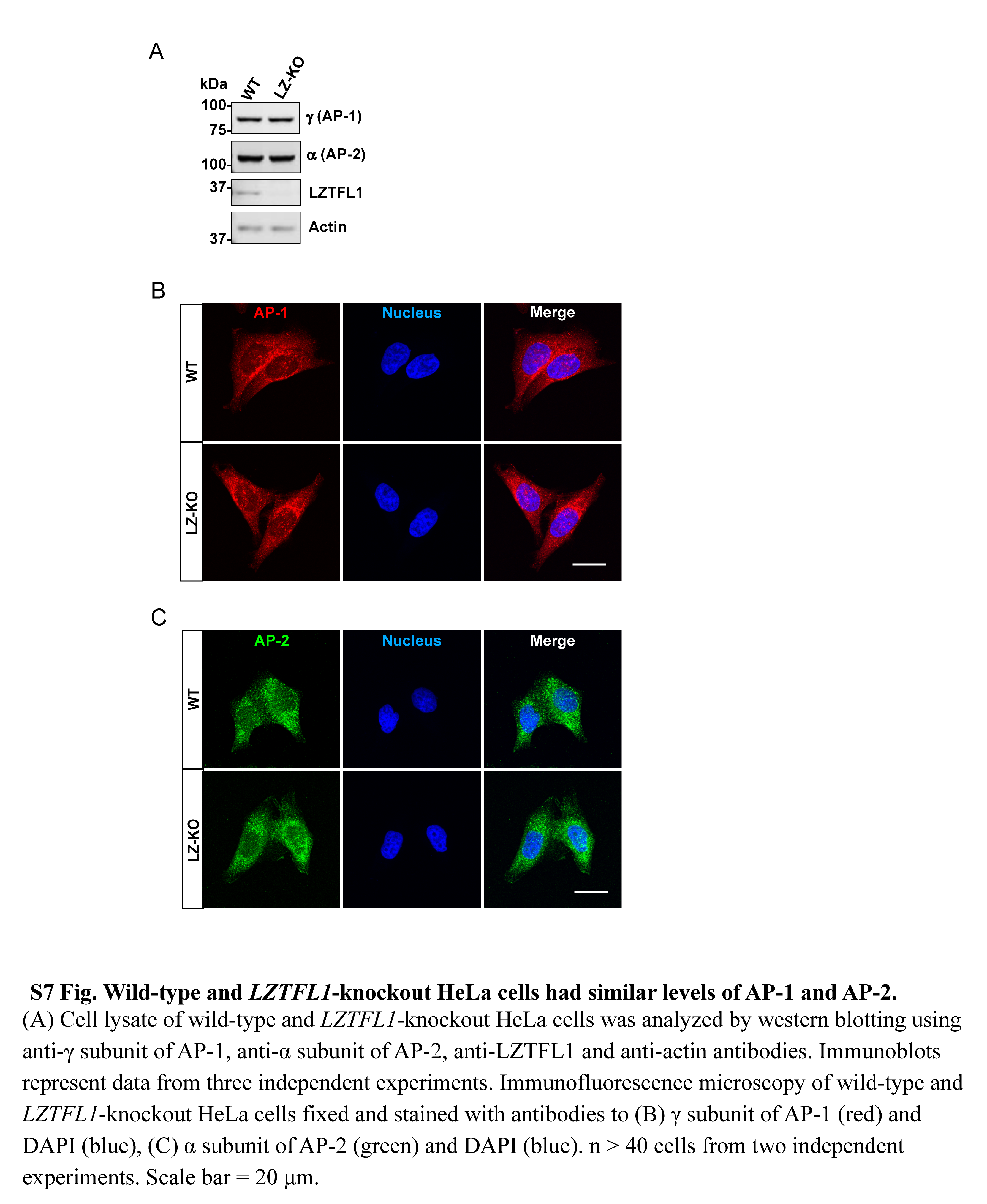

Supplement: S7 Fig — (A) Cell lysate of wild-type and LZTFL1-knockout HeLa cells was analyzed by western blotting using anti-γ subunit of AP-1, anti-α subunit of AP-2, anti-LZTFL1, and anti-actin antibodies. Immunofluorescence microscopy was conducted on wild-type and LZTFL1-knockout HeLa cells fixed and stained with antibodies to (B) γ subunit of AP-1 (red) and DAPI (blue) and (C) α subunit of AP-2 (green) and DAPI (blue). n>40 cells from two independent experiments. Scale bar = 20 μm. (TIF) [file pone.0226298.s007.tif]

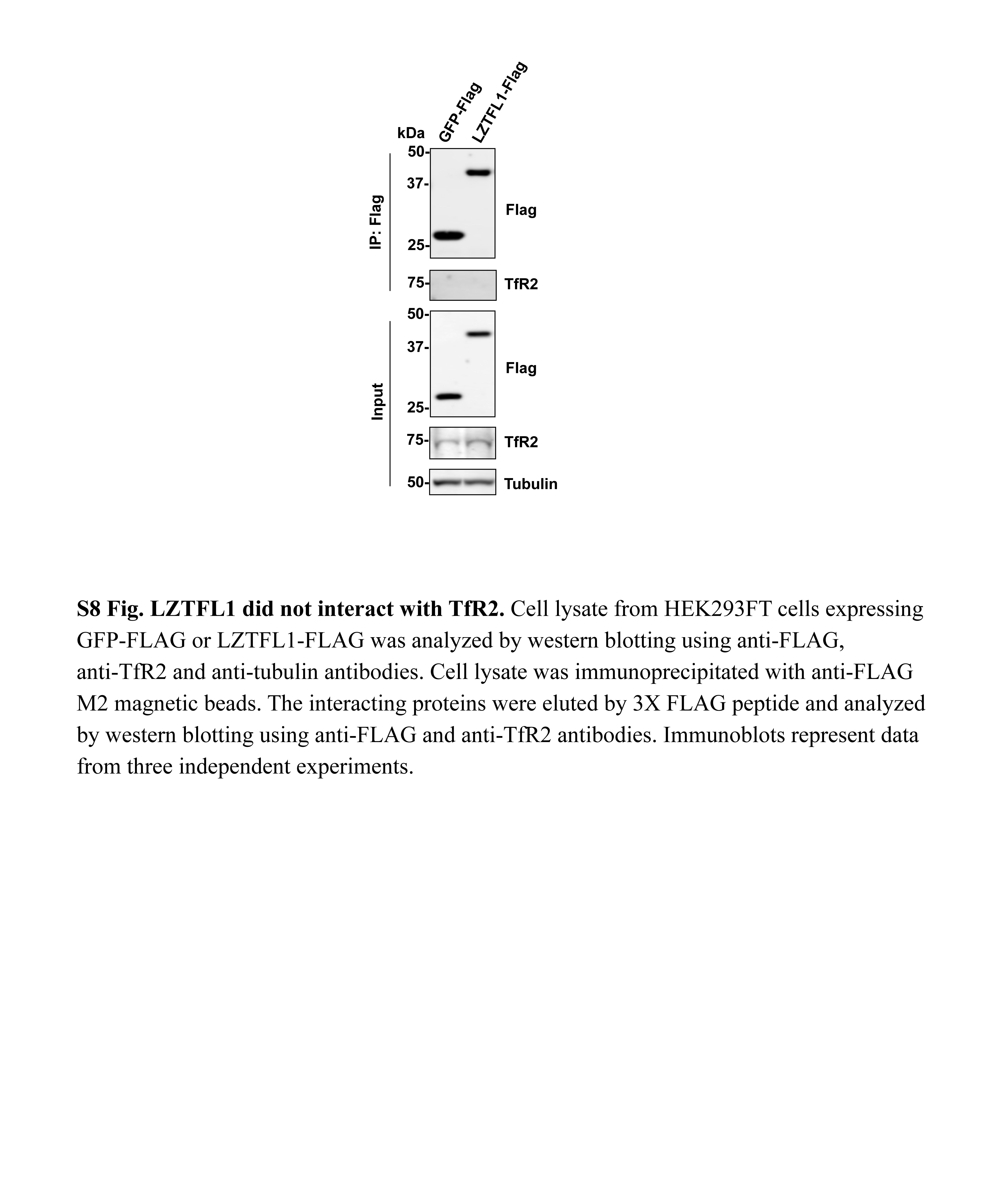

Supplement: S8 Fig — Cell lysate from HEK293FT cells expressing GFP-FLAG or LZTFL1-FLAG was analyzed by western blotting using anti-FLAG, anti-TfR2 and anti-tubulin antibodies. Cell lysate was immunoprecipitated with anti-FLAG M2 magnetic beads. The interacting proteins were eluted by 3X FLAG peptide and analyzed by western blotting using anti-FLAG and anti-TfR2 antibodies. (TIF) [file pone.0226298.s008.tif]

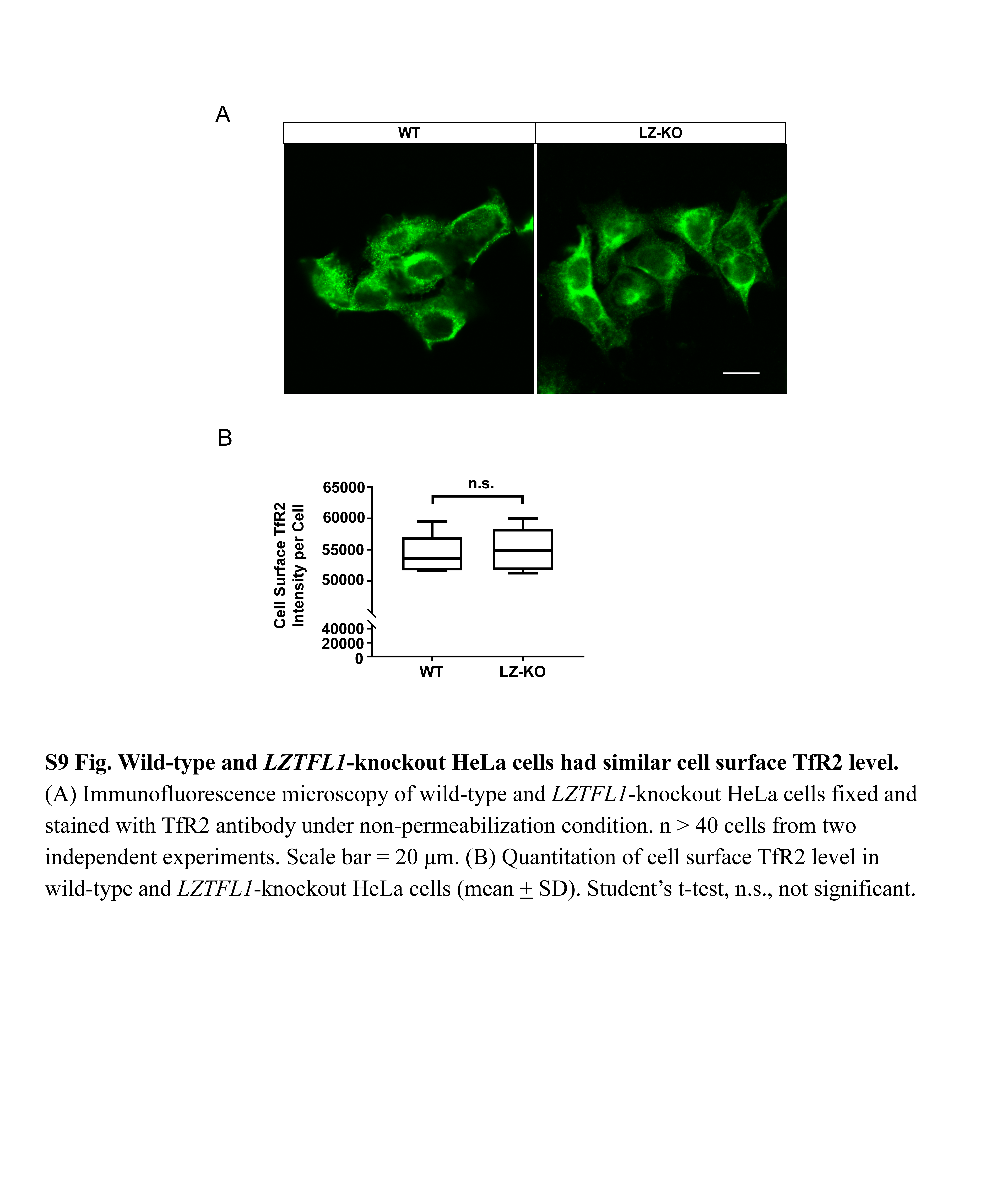

Supplement: S9 Fig — (A) Immunofluorescence microscopy of wild-type and LZTFL1-knockout HeLa cells fixed and stained with TfR2 antibody under non-permeabilization condition. n > 40 cells from two independent experiments. Scale bar = 20 μm. (B) Quantitation of cell surface TfR2 level in wild-type and LZTFL1-knockout HeLa cells (mean ± SD). Student’s t-test, n.s., not significant. (TIF) [file pone.0226298.s009.tif]

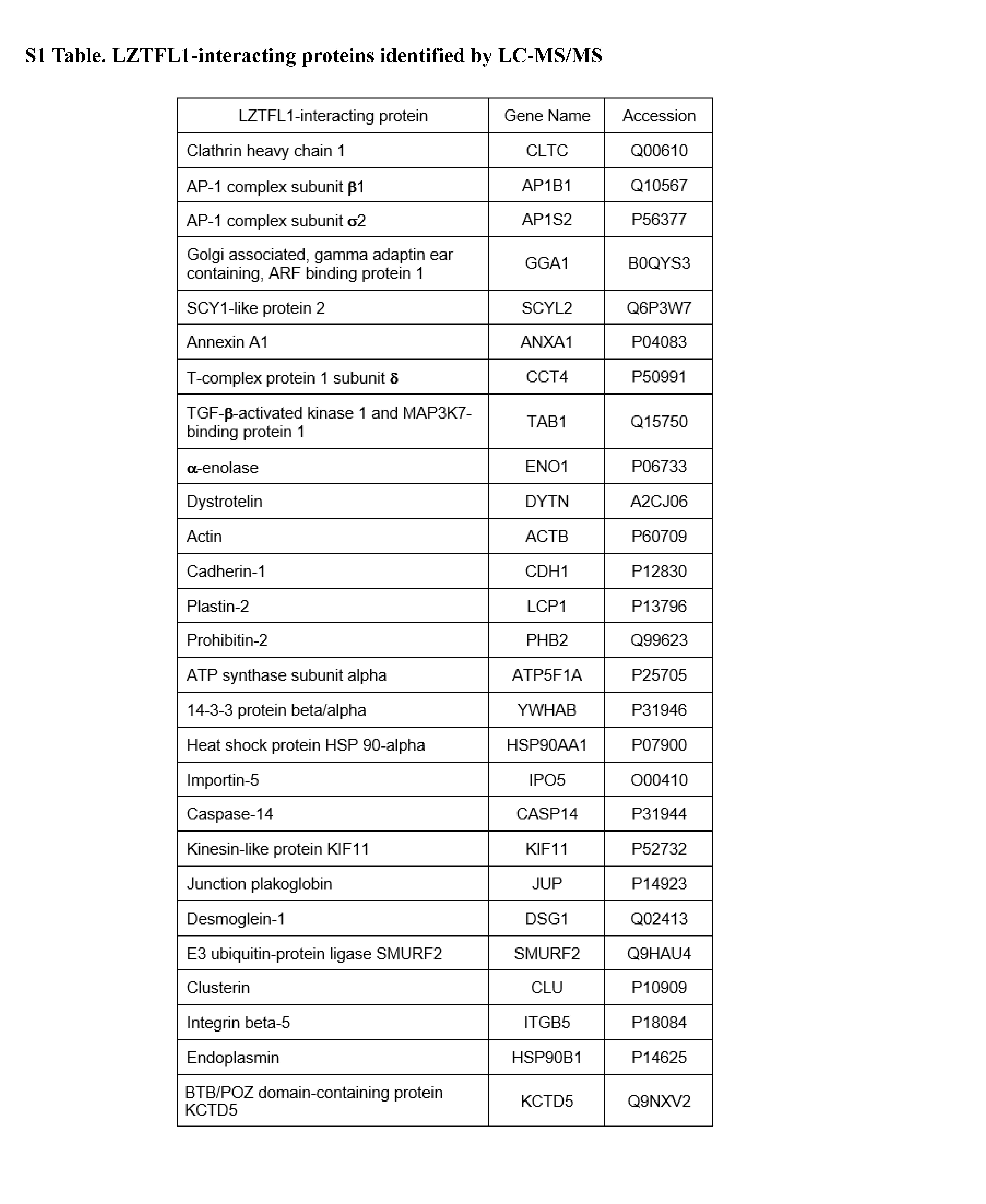

Supplement: S1 Table — (TIF) [file pone.0226298.s010.tif]

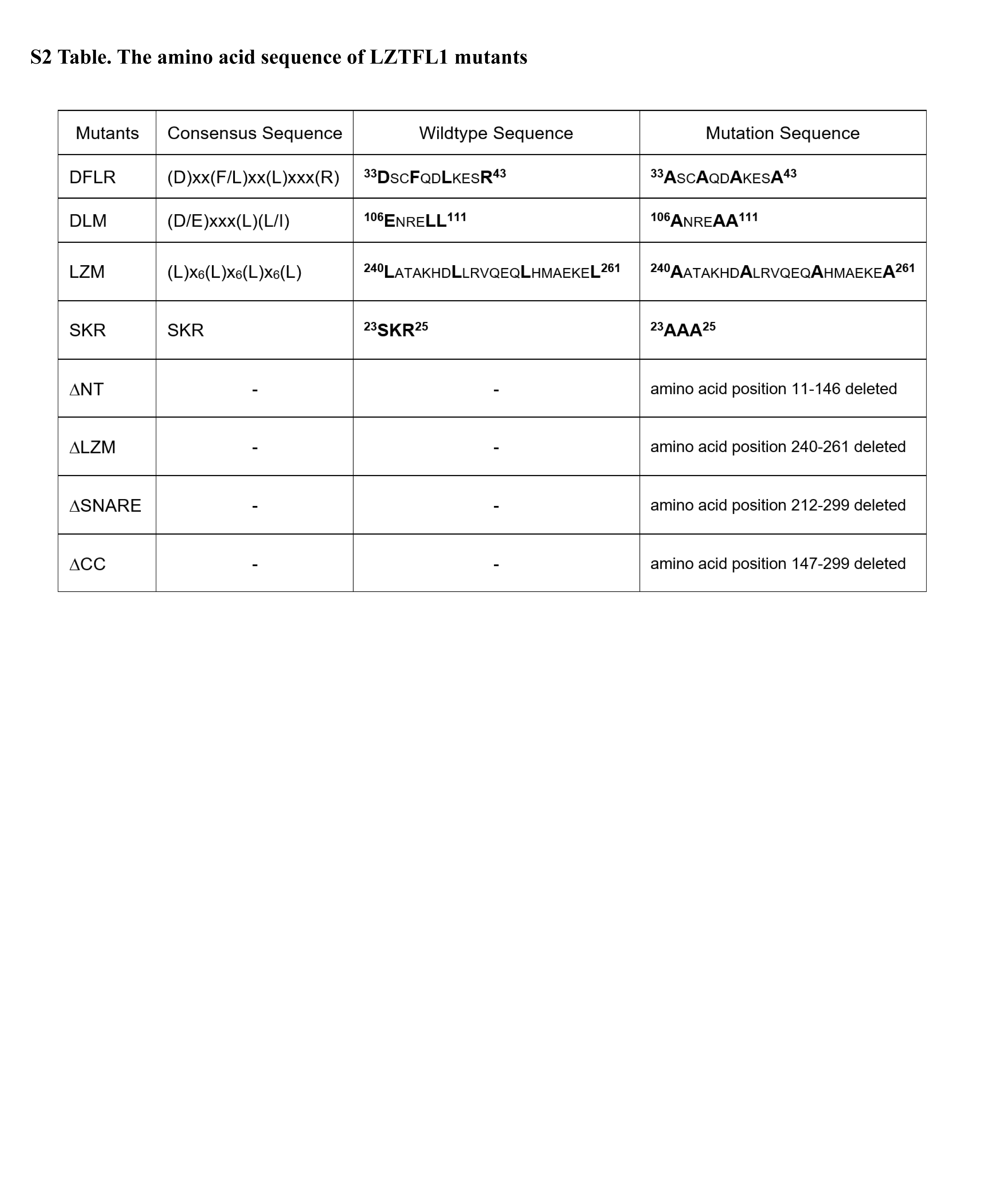

Supplement: S2 Table — (TIF) [file pone.0226298.s011.tif]
